# In Pursuit of a Better Biocide Composition: Synergistic and Additive Effects of QAC-Based Formulations Against Planktonic and Biofilm Cultures

**DOI:** 10.3390/ijms262412098

**Published:** 2025-12-16

**Authors:** Nikita A. Frolov, Mary A. Seferyan, Elena V. Detusheva, Elizabeth Son, Ilya G. Kolmakov, Anatoly N. Vereshchagin

**Affiliations:** 1 N. D. Zelinsky Institute of Organic Chemistry, Russian Academy of Sciences, Leninsky Prospect 47, 119991 Moscow, Russia; nfrolov@ioc.ac.ru (N.A.F.); marysev@ioc.ac.ru (M.A.S.); detushevaev@obolensk.org (E.V.D.); ikolvilya@outlook.com (I.G.K.); 2 State Research Center for Applied Microbiology and Biotechnology, City District Serpukhov, Moscow Region, 142279 Obolensk, Russia; e_swon2001@mail.ru; 3 Faculty of Chemistry, Lomonosov Moscow State University, Leninskie Gory, 1-3, 119991 Moscow, Russia

**Keywords:** cationic biocides, QACs, antibacterials, antibiofilm agents, synergism, antiseptic compositions, multi-resistant bacteria, cross-resistance, ESKAPEE, checkerboard assay

## Abstract

Managing bacterial infections and the spread of microbial resistance is one of the most critical and complex tasks of modern healthcare infrastructures. Antiseptics and disinfectants such as biocides play a significant role in controlling microbial resistance by reducing the microbial load on surfaces, skin, and environments, thereby limiting the opportunity for pathogens to proliferate and develop resistance. Herein, we tested the different interactions of quaternary ammonium compound (QAC)-based biocide compositions in pursuit of a better antimicrobial performance. An extensive microbiological analysis was conducted for 12 selected compositions of various combinations of mono-QACs, bis-QACs, and alcohols on 17 strains of bacteria of the ESKAPEE group and fungi, including 11 clinical highly resistant varieties, highlighting synergistic or additive dynamics. The evaluation showed noticeable improvements in activity, with up to 16-fold MBC and 32-fold MBEC reductions for alcohol-based compositions of lead QAC. Moreover, synergistic interactions were detected and confirmed via an optimized checkerboard assay for pyridinium QAC combinations against planktonic Gram-positive *S. aureus* with a fractional inhibitory concentration index (FICI) and fractional bactericidal concentration index (FBCI) of 0.39–0.5 and Gram-negative *A. baumannii* biofilms. The studied biocides demonstrated the long-term preservation of antimicrobial efficiency without resistance development during a 40-day period and do not induce QAC-associated cross-resistance for four commercially available antibiotics with similar mechanisms of action.

## 1. Introduction

The control of bacterial spread faces significant challenges, particularly due to the rise in microbial resistance and complex transmission pathways [1]. Overprescription in healthcare and widespread biocide misuse accelerate resistant-gene spread [2]. Biological adaptation through enzymatic antibiotic inactivation, target modification, efflux pumps, and horizontal gene transfer allows bacteria to harbor resistant genes in wastewater systems, healthcare facilities, medical equipment, and various surfaces [3,4,5,6,7]. Addressing these crucial threats requires coordinated biocide administration, improved sanitation infrastructure, and innovation in both antimicrobial therapies and antimicrobial synthesis.

One of the main fundamental elements for controlling the spread of bacterial infections is the timely use of antiseptics and disinfectants. Although they are both chemical agents whose primary function is to destroy or inhibit microorganisms, they differ principally in their application and safety profiles. While antiseptics are applied to living tissues such as skin or mucous membranes to reduce or inhibit microbial growth, disinfectants are only used on inanimate objects and surfaces to eradicate microorganisms [8,9,10,11]. Both antiseptics and disinfectants contain various active chemical agents (biocides) with broad-spectrum antimicrobial activity, of which, one of the common classes include alcohols [12,13,14,15,16] and quaternary ammonium compounds (QACs) [17,18,19,20]. They are often formulated with each other to improve overall the antimicrobial efficacy and reduce the required contact time for disinfection. Our previous study showed that combining QACs with alcohols could result in a 32-fold MIC and 16-fold MBC reduction compared to using QACs alone [21].

Another approach to increasing the effectiveness of antimicrobial formulations is antibiotic and/or biocide synergism. The synergistic action of antibacterial agents involves combining two or more antimicrobial compounds to potentiate activity, improve resistance management, and reduce toxicity compared to individual use. This approach leverages complementary mechanisms to achieve greater antimicrobial potency and delay resistance development [22,23,24,25,26,27]. Synergism in disinfectant and antiseptic formulations, including QACs, have been investigated for a prolonged period, and research continues to this day [28,29,30,31,32,33,34,35]. However, while many products claim to exhibit synergistic interactions, some scientific data indicates that such effects are rare and context-dependent, whereas additive interactions are more common [36]. These are attributed to broad and nonspecific membrane-targeting mechanisms of action, limiting opportunities for enhanced synergy. While the possibility of discovering synergistic effects remains an attractive topic to the scientific community and in the commercialization of the resulting products, it is important to maintain experimental integrity and stay within the correct terminology to avoid the inappropriate attribution of additive and indifferent effects to synergism.

**Figure 1:**
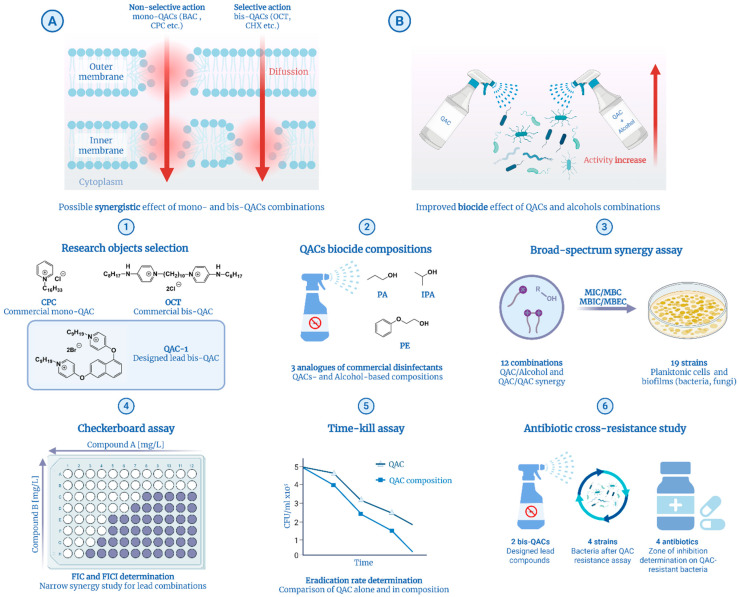
Main concepts (**A**,**B**) and workflow (**1**–**6**) of the current study. Concepts: (**A**) Mechanistic differentiations of mono- and bis-QACs proposed by Sanchez et al. [37]. (**B**) Synergistic effect of QACs and alcohols against bacteria. Workflow: (**1**) Selection of research objects including commercial and developed antimicrobial agents. (**2**) Preparation of biocidal compositions of antiseptic and disinfectant analogs, as well as combinations of QACs. (**3**) Broad microbiological synergy study on ESKAPEE pathogens and fungi. (**4**) Checkerboard assay for confirmation of synergy for lead QAC combinations. (**5**) Time–kill kinetics assay of lead compounds and compositions. (**6**) QAC-associated antibiotic resistance study via a combination of the adaptive evolution and disk diffusion methods. Created in BioRender. Seferyan, M. (2025) https://BioRender.com/lbgkurz.

Herein, we carried out a study to evaluate the different effects of QAC-based formulation components on overall antimicrobial activity profiles. The present work is based on two main proposed concepts. Firstly, beneficial QAC and alcohol interactions lead to improved QAC solubility and boosted antimicrobial activity. The theory is based on previously conducted studies which often used antiseptic and disinfectant compositions, containing both QACs and alcohols. Secondly, the possibility of synergistic interaction of mono- and bis-QAC derivatives toward Gram-negative bacteria disruption. A recent report by Sanchez and colleagues suggested novel mechanistic insights for bis-QACs’ antibacterial actions. The theory proposes a mechanistic selectiveness in bis-QAC as well as bis-QPC (quaternary phosphonium compound) actions toward the inner phospholipid membrane, whereas mono-QACs are unselective and target both the outer and inner layers [37]. Therefore, we suggest that these two classes of biocides could work in synergy, targeting both membranes simultaneously. To our knowledge, there are no previously published studies regarding this topic. Moreover, we touched on the topic of widespread concerns about QAC-associated antibiotic resistance. The current study’s outline and work concepts are depicted in Figure 1. Numerous developments in the biocide field are currently being published; however, unfortunately, there is a significant gap between scientific research and the introduction of these agents into real-world clinical practice [38]. The present study will be useful both from a fundamental perspective, as it sheds light on the synergistic effect between mono- and bis-QACs, and from a practical standpoint, as we are investigating formulations that are suitable for commercialization. Thus, we hope that our research will help promote the integration between fundamental research and the practical implementation of biocide development and aid with the design of better antimicrobial compositions.

## 2. Results and Discussion

### 2.1. Basic Concepts of QAC Synthesis and the Biocidal Activity of QAC-Based Compositions

**Scheme 1:**
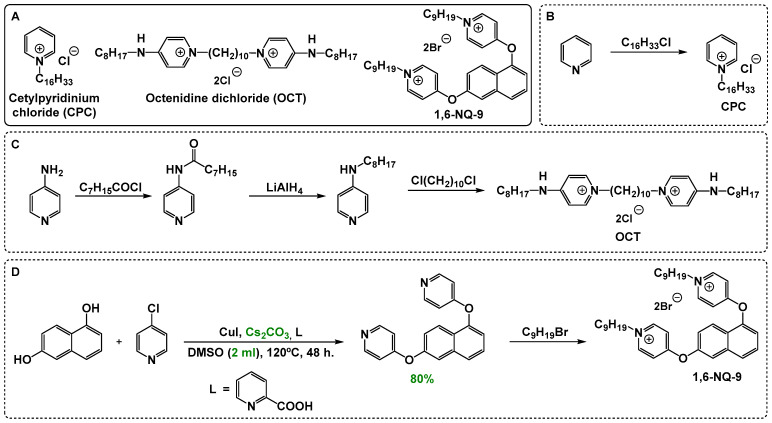
Main compounds selected for the microbiological evaluation of QAC-based compositions (**A**) and the basic concepts of mono- (**B**) and bis-QAC (**C**,**D**) synthesis. Optimized reaction conditions are highlighted in green.

As the main objects for the study, we selected two commercial biocides including mono-QAC cetylpyridinium chloride (**CPC**) and bis-QAC octenidine dihydrochloride (**OCT**), as well as the leading compound from our previous laboratory work, dioxynaphthalene-derived **QAC 1** (Scheme 1A). The selected panel of compounds includes both widely used biocides of various subclasses of QACs and those not yet fully studied. Moreover, the presence of structural uniformity (pyridinium cores and alkyl chains) and similar physicochemical properties (surface-active properties) of the molecules further ensure experimental integrity.

Since the central purpose of this work is to evaluate the possibility of the combined action of different biocides, it is important to understand what benefit can be derived from variable types of interactions, specifically, whether the combined effect exceeds the sum of their individual effects (synergistic), is equal to the sum (additive), or is less than the sum (antagonistic). While the revelation of a synergistic effect is usually a positive outcome for antibacterial agents and that of antagonism is negative, the usefulness of an additive interaction is dependent on several factors. Among others, a crucial aspect when evaluating the use of a combination of antiseptics or disinfectants, which are not generally intended for internal use, is the availability and low cost of synthesis.

QACs are synthesized mainly through quaternization reactions involving tertiary amines or other similar nitrogen moieties and alkyl halides, with structural variations tailored to optimize antimicrobial activity. While the synthesis of mono-QACs is usually straightforward and achieved from readily available scaffolds, bis-QACs often require the preliminary synthesis of starting materials with two amine cores or core-tail blocks. For example, **CPC** is synthesized through simple N-alkylation of pyridine (Scheme 1B), whereas **OCT** is acquired by linking two alkylaminopyridines with 1,10-dichlorodecane (Scheme 1C). Thus, **OCT** is less affordable, as it requires a preliminary 2–3 stage synthesis of alkylaminopyridines [39]. Therefore, even the presence of an additive effect between these QACs will reduce the cost of production for the biocidal composition while maintaining a high level of antimicrobial action. The lead compound **1,6-NQ-9** studied here was synthesized through a simple two-step method including Ullmann-type condensation and N-alkylation [40] (Scheme 1D). Herein, we proposed an alternative variant of condensation with caesium carbonate as a base in a significantly reduced volume of DMSO (2 mL compared to 50 mL) and at a lower temperature (120 °C compared to 140 °C). The reaction procedure was optimized for bis-scaffolds from ref. [41].

As the main components of the study, we selected 12 compositions of various combinations of mono-QACs, bis-QACs, and alcohols, including propanol-1, isopropanol, and phenoxyethanol, consistent with prior successful findings and commercial analogs for disinfectants [21,42,43]. Alcohol-based mixtures were prepared in the following formulations: QAC + phenoxyethanol/water (solution 2%, *v*/*v*) similar to the drug “Octenisept” (Schülke & Mayr)—**PhE**; QAC + isopropyl alcohol/water (solution 63/5%, *v*/*v*)—**IPA**; QAC + isopropyl alcohol/propyl alcohol/water (1/2/2, *v*/*v*/*v*)—**IPAP**.

An extensive microbiological analysis of the obtained mixtures was carried out on 17 strains of bacteria of the ESKAPEE group and fungi, including 11 clinical highly resistant varieties. The investigated pathogens were categorized into two groups: reference strains and clinical isolates. Reference strains are a standard panel of ATCC cell line microorganisms from the ESKAPEE group, including *S. aureus* ATCC 43300, *E. coli* ATCC 25922, *K. pneumoniae* ATCC 70060, *A. baumannii* ATCC 15308, and *P. aeruginosa* ATCC 27853. In addition, the reference strains included the yeast fungi *C. auris* DSM 21092. *C. auris* is an emerging multidrug-resistant pathogen with distinct biological and pathogenic traits compared to *C. albicans*, exhibiting lower virulence but higher persistence and transmission potential in healthcare settings [44,45]. Clinical isolates are multidrug-resistant bacterial strains that were isolated in cases of infection in hospitalized patients in 2018–2021, including *E. coli* B-3421/19, *E. coli* C226691/21, *K. pneumoniae* B-2523/18, *K. pneumoniae* C24540/21, *S. aureus* B-8648, MRSA 0576, *P. aeruginosa* B-2099/18, *P. aeruginosa* C23520/21, *A. baumannii* B-2926/18, *A. baumannii* C23382/21, and *C. auris* KA9. The analysis was carried out both on planktonic colonies of microorganisms and biofilm forms, allowing for the determination of five types of antimicrobial action indicators: minimum inhibitory concentration (MIC), minimum bactericidal concentration (MBC), minimum fungicidal concentration (MFC), minimal biofilm inhibition concentration (MBIC), and minimal biofilm eradication concentration (MBEC). The values of the initial activity of individual components, including alcohol systems, and **OCT**-based compositions were determined earlier [21] and are given for comparison.

Firstly, biocide action against planktonic bacterial cultures were studied (Table 1). The evaluation showed noticeable improvements in the activity of QAC-based alcohol compositions on some Gram-negative strains, especially *K. pneumoniae* ATCC 70060, *A. baumannii* ATCC 15308, and *A. baumannii* B-2926/18. Thus, an up to 16-fold MBC reduction was registered for the **1,6-NQ-9** and **CPC** mixtures. Almost no visible changes were detected for *E. coli* ATCC 25922 and *K. pneumoniae* B-2523/18, where only **1,6-NQ-9** showed modest differentiations in propanol combinations. Solely **OCT**-based mixtures demonstrated decent spikse in activity on both reference and clinical *P. aeruginosa,* which was described earlier [21]. In contrast, the biocidal effect against *S. aureus* increased significantly only for **CPC** and **QAC-1**. Notably, the usage of a combination with alcohols allowed mono-QACs to achieve activity levels close to bis-QACs against some pathogens, confirming the effectiveness of this approach. A surge of activity can occur because alcohols significantly enhance drugs’ solubilities by interacting with both hydrophobic tails and the charged moieties of QACs. Furthermore, solvation could improve the membrane’s permeability, improving QACs’ performances. While improvements in QAC activity were seen for all alcohol systems, the **IPAP** combination appeared to be the most effective, with nearly half of the efficacy values increasing by two or more dilutions. Interestingly, with the increase in the activity of the alcohol mixtures, the effect was manifested both for bacteriostatic and bactericidal action in most cases (almost 80% of the total). However, the increase in the bactericidal effect was more pronounced, which is advantageous when using biocidal agents on surfaces. This effect can be due to the alcohol’s mechanism of action on the bacterial cell. Alcohols are generally considered to exert a bactericidal effect rather than a bacteriostatic effect, causing the denaturation and coagulation of proteins within microbial cells, which disrupt cell membranes and ultimately result in cell death [8,46].

Next, we conducted a study of the effect of antiseptic compositions on freshly formed biofilm cultures. Although alcohols are not considered to be antibiofilm agents; they have, on the contrary, been shown to increase the formation of biofilms by bacteria like *S. aureus* and *S. epidermidis* at formulations commonly used in medical settings [47]. We were able to detect a strong increase in activity on all pathogens for both mono- and bis-QAC combinations (Table 2). The most prominent effect was observed on Gram-negative *A. baumannii* ATCC 15308 and *K. pneumoniae* B-2523/18, with an up to 16-fold MBEC reduction, as well as *K. pneumoniae* ATCC 70060, with a 32-fold MBEC reduction for **IPAP 1,6-NQ-9**. Even on the highly resistant *P. aeruginosa* B-2099/18 strain, a 64-fold MBIC and 32-fold MBEC decrease was recorded for **QAC-1**. *P. aeruginosa* biofilms are recognized as one of the most life-threatening bacterial forms and pose a major challenge in clinical treatment worldwide [48]. Moreover, obtained compositions showed good MBIC/MIC and MBEC/MBC ratios within a 0.5–2 range in almost 60% of cases, indicating a one dilution difference within planktonic cells and biofilms. In the same way as on planktonic cells, the **IPAP** combination was more effective against biofilms among the studied mixtures. It could be proposed that the observed spike of antibiofilm activity occurred via the alcohol-associated denaturation of proteins in biofilm matrix [49], which allowed QACs to reach the bacteria within the biofilm and act more efficiently in lower concentrations. Another important factor could be the better solubility of QACs in alcohol–water as compared to in DMSO–water mixtures, which could prevent QACs from excessive sedimentation during mixing with a suspension of bacteria in broth, therefore boosting activity.

A microbiological assay against five additional multi-resistant bacterial strains from clinical samples during the COVID-19 period in 2021 showed similar results, with activity improvement predominantly on Gram-negative strains and biofilms (Table S1). It is worth noting that a boost in bactericidal action was also evident for both planktonic and biofilm cultures. Nonetheless, no considerable contrast was exhibited between the types of microbial organization when analyzing the antifungal activity on *C. auris* strains (Table S2). Surprisingly, in this case the effect was more pronounced for mono-QACs. Thus, **IPAP CPC** and **IPA CPC** outperformed the corresponding combinations with **1,6-NQ-9**.

### 2.2. QAC Combinations Synergy Test

**Figure 2:**
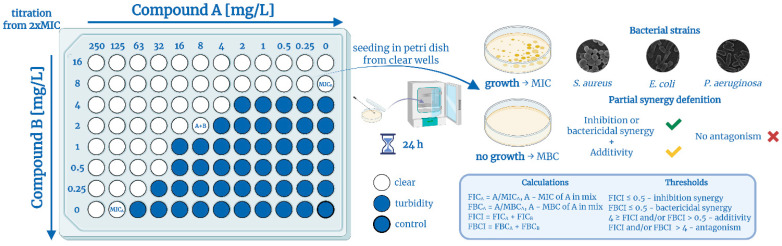
Checkerboard assay and partial synergy concepts. Created in BioRender. Seferyan, M. (2025) https://BioRender.com/p5qz6o1.

Firstly, for the QAC + QAC synergy test, QACs were mixed in advance in a 1:1 ratio by mass concentration to facilitate the experiment and initial calculations for primary screening on a large panel of 17 microorganisms, listed above. Although this only measures the antimicrobial efficacy of compounds in a single fixed ratio (11 combinations), it is a cost-effective and time-efficient approach that allows for an initial assessment of synergistic potential across a broad spectrum of pathogens, including biofilms. Secondly, to confirm the possible synergy, we performed the checkerboard assay (Figure 2). This method is widely used to analyze the combined antimicrobial effect of systems containing two active components via a two-dimensional grid arrangement [50,51]. The main advantages of this method are the simultaneous evaluation of the effectiveness of multiple ratios of each pair of substances (77 combinations) around the optimal activity range and a visual comparison with the current MIC. The detailed methodology is given in the experimental section.

In most cases, fractional inhibitory concentrations (FICs) and fractional inhibitory concentration indices (FICIs) are calculated for the further interpretation of the effects of biocides combinations. When the FICI is less than 0.5, the interaction of substances is taken to be synergistic, and when the FICI is more than 4, it is taken to be antagonistic [52,53]. At the same time, the interpretation for the interval 0.5–4 differs. Several main options can be found in the following examples: (1) 0.5–4—indifference [33]; (2) 0.5–1—partial synergy, 1–2—additivity, 2–4—indifference [24,27]; (3) 0.5–1—partial synergy, 1–4—indifference [32]; and (4) 0.5–1—additivity, 1–4—indifference [23,34,36]. It is worth noting that the differences between “partial synergy” and “synergy” remain unclear. In this paper, we propose an optimized version of the experiment with an alternative interpretation that allows the term “partial synergy” to be valid for a certain experimental result.

**Figure 3:**
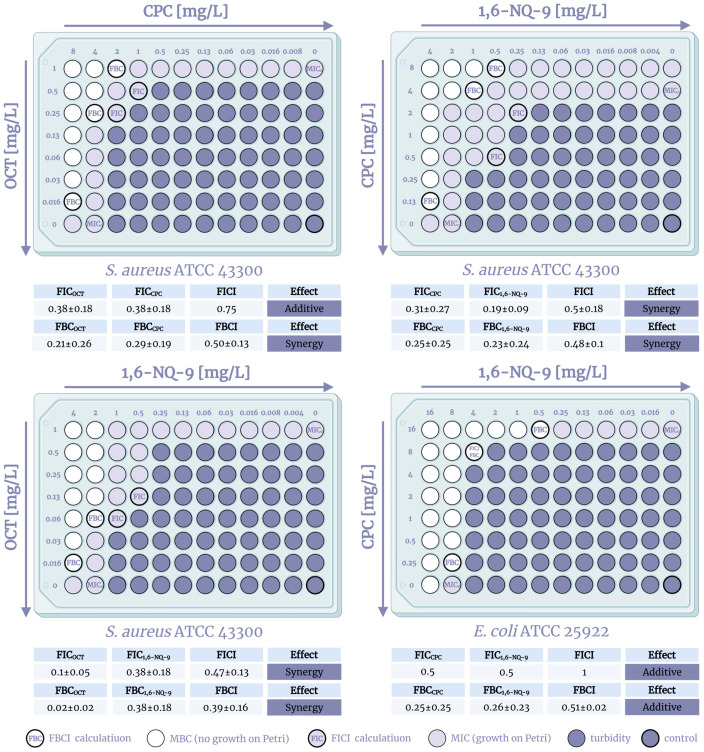
Visualization of the checkerboard assay, FICI, and FBCI calculations on *S. aureus* ATCC 43300 and *E. coli* ATCC 25922. Created in BioRender. Seferyan, M. (2025) https://BioRender.com/rwps219.

As noted above, most microbiological studies use MIC as the gold standard for determining the effectiveness of antibacterial agents. However, we are convinced that this paradigm is not relevant when discussing biocides used to eradicate microorganisms. The main value in this case should be the MBC—a concentration needed to kill 99.9% of the colony of microorganisms and the capacity of substances for bactericidal action. When combining the determination of MIC and MBC in a primary screening test and a checkerboard assay, it is possible to interpret the results by an additional indicator—the fractional bactericidal concentration index (FBCI). Combining these two indicators will allow for two definitions of synergy—inhibitory and bactericidal. Thus, in the presence of both synergy options, the interaction can be considered a complete synergy, and in the presence of only one—a partial synergy (Figure 3). The same principle can be applied to determine the antibiofouling effect. Fractional indices for five types of antimicrobial activity against the 17 strains acquired during the primary screening of 1:1 QAC combinations are represented in Table 3 and Table S3. Our rationale for selecting the fractional indices threshold criteria is based on the standard error metric for the microdilution method, where the permissible deviation is considered to be a difference of one dilution. Thus, taking into account the recalculation of the indices, synergism will be observed at a value of 0.5 and lower. The range of 0.5–4 was divided into two main parts: additivity (0.5–2), when the combined effect of two substances equals the sum of their independent contributions, and indifference (2–4), when mixing the substances does not affect their activity level. We believe that these two types of action cannot be attributed to the same nature, since indifference does not carry any potential benefit, unlike additivity (see Section 2.1). If the standard error is removed from the calculation, additivity will be observed only at an index value of 1.

Expectedly, the evaluation showed that the QACs’ combined effect is mostly additive in nature with more than half of the indices in the 0.5–2 range. Thus, only 20% of the compositions displayed full synergy, mainly due to the pronounced effect on *A. baumannii* strains, where indices reached up to 0.03 (the equivalent of a 64-fold MBC reduction), expressing strong synergy. An unexpected result was the evidence of a possible synergistic effect against Gram-positive *S. aureus* ATCC 43300, where the combination of **CPC + 1,6-NQ-9** demonstrated synergism against both planktonic cells and biofilms with fractional indices of 0.13–0.38. Partial synergism was observed in 40% of the studied cases. However, for QACs, bactericidal synergy is far more relevant than bacteriostatic, since it may be more applicable to the real conditions of biocide use. Partial bactericidal synergism was observed on five strains of planktonic cells, including *S. aureus* ATCC 43300, and on six biofilms. It is worth noting that no cases of complete synergy were recorded for the fungal pathogen *C. auris,* and two-thirds of the interactions were additive (Table S4).

**Figure 4:**
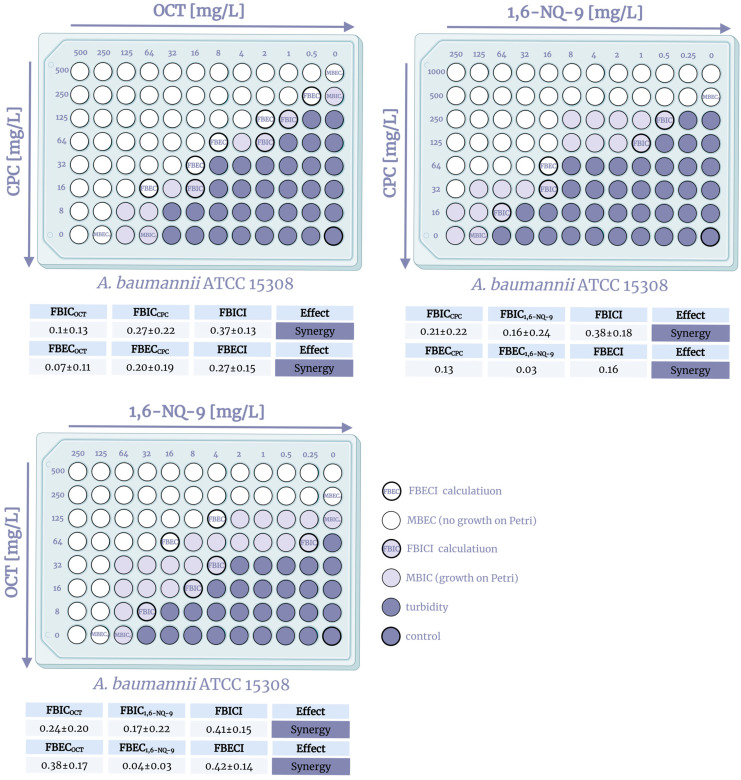
Visualization of the checkerboard assay, FBICI, and FBECI calculations on *A. baumannii* ATCC15308. Created in BioRender. Seferyan, M. (2025) https://BioRender.com/2h5jbyc.

Next, to confirm the outcome of the prior screening of synergistic interactions, the checkerboard method was carried out using three combinations of QACs on three pathogens, *S. aureus* ATCC 43300, *E. coli* ATCC 25922, and *A. baumannii* ATCC 15308, in cases of complete or partial synergism. A graphic visualization and the calculations of the experiment are presented in Figure 2, Figure 3 and Figure 4. The methodology of this research is described in detail in the experimental section. To calculate the fractional indices, the MIC_0_ were used, since the MIC_0_ obtained in the checkerboard method did not differ from the initial ones by more than one dilution, which is taken into account in the standard error when correlating the fractional indices. The checkerboard assay was conducted in triplicates and visualized as the mean values.

The study of combinations of QACs on the *S. aureus* strain confirmed the presence of a synergistic antibacterial effect of the components. Moreover, both combinations of mono- and bis-derivatives and bis-derivatives with each other were effective. It is especially worth noting the lower FBCI (0.39–0.5) compared to FICI (0.47–0.75), which indicates the prevalence of increased bactericidal action. The observed effect arises from the fact that QACs are mostly considered bactericidal agents due to their membrane-lytic properties, while bacterial growth is usually reduced through protein synthesis inhibition while maintaining membrane integrity. Unfortunately, an additive effect was recorded against Gram-negative *E. coli,* where the fractional indices were 0.51–1. However, as noted above, with a combination of mono- and bis-QACs, additivity may be sufficient enough, provided that the amount of the bis-derivative used, which is more expensive to produce, is reduced. In the case of *A. baumannii*, similar results were discovered with the exception of the **1,6-NQ-9** and **CPC** combination, where partial synergy was noted with an FBCI = 0.49 (Figure S1). The obtained picture allows us to assume that the possible different mechanisms of action of mono- and bis-QACs on the dual membranes of Gram-negative bacteria do not induce a synergistic effect when designated QACs are used in combination on planktonic cultures. Nevertheless, we were able to detect and confirm synergism when using combinations of pyridinium QACs on Gram-positive *S. aureus* and a possible synergy against biofilms, which is encouraging for further studies.

Therefore, we conducted an additional checkerboard assay on biofilm cultures of *A. baumannii* (Figure 4). Surprisingly, all combinations showed full synergy, demonstrating increased biofilm inhibition as well as biofilm eradication. The mechanism of biofilm eradication is a complex process that additionally involves interfering with various stages of biofilm formation and maturation, such as inhibiting initial bacterial adhesion to surfaces, disrupting cell-to-cell communication via quorum sensing, modulating signaling molecules like c-di-GMP, or degrading the biofilm’s extracellular matrix and mature biofilm structures [54,55,56]. Given that intermolecular synergy was observed for already formed biofilms, it can be speculated that enhanced activity may be associated with the breakdown of biofilm matrix components such as extracellular DNA, proteins, or polysaccharides. It is acknowledged that QACs can induce DNA and protein damage [57,58,59]. However, this hypothesis requires further research, which is outside the scope of the present study. Furthermore, the constraints of the checkerboard method embrace inherent instability in dilution steps, labor-intensive manual setup, and difficulties in interpretation. While it provides useful data on interactions, it lacks information on time-dependent effects.

### 2.3. Time–Kill Kinetics Assay

**Figure 5:**
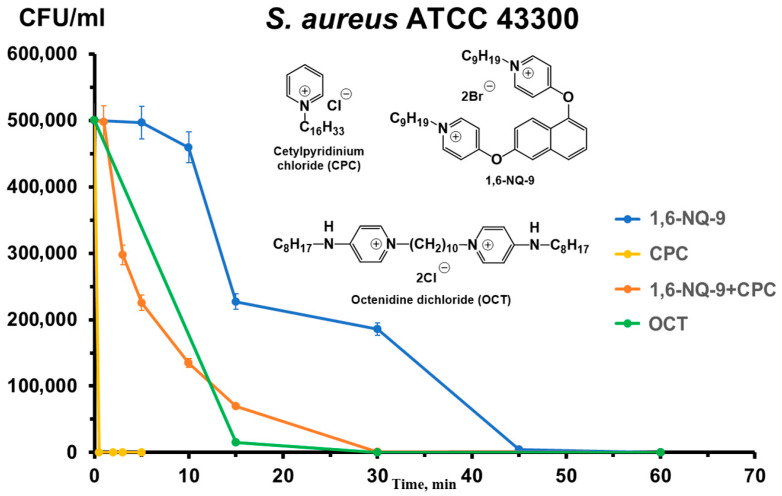
Time–kill dynamics of mono- and bis-QAC combinations.

In addition to the level of antibacterial activity, another important indicator of biocide effectiveness is the speed of its action on a bacterial colony. The combination of action time and activity helps to determine for which profile of biocides the agent under study is more suitable. In the case of QACs, this usually refers to their use as an antiseptic and/or disinfectant. Moreover, for the purposes of this study, a comparison of the speed will also indicate the difference in the antibacterial profiles of QACs in combination and separately. This indicator is assessed using the widely used time–kill kinetics assay [60,61]. The reference strain of the planktonic culture *S. aureus* ATCC 43300 was used as the object of the study due to the confirmed synergy of the **1,6-NQ-9** and **CPC** combination (Figure 5).

The results revealed interesting correlations between the rate of action of the substances and their structure. **CPC** exhibited a rapid antibacterial effect, eradicating a colony of the selected pathogen within 3 min, whereas the combination of **1,6-NQ-9** and **CPC** required 20–30 min to achieve the same effect. This interaction may be due to the lower solubility of bis-QACs in complex culture systems, which reduces the bioavailability of the composition, delaying the bactericidal effect. We observed a similar pattern previously [62].

### 2.4. Bacterial Resistance Assay

**Figure 6:**
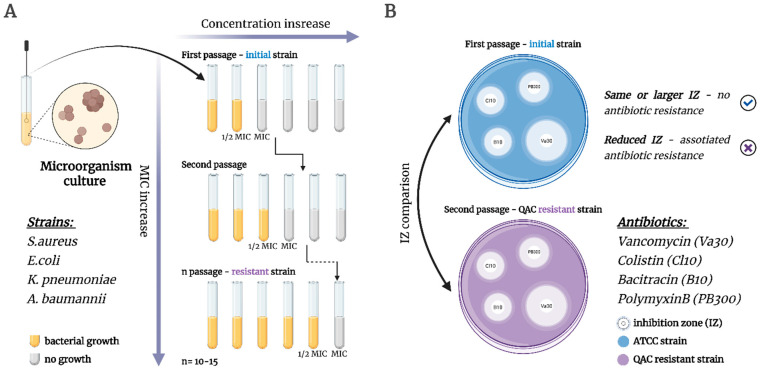
Workflow of QACs and antibiotics resistance assay. (**A**) The selection of QAC-resistant microorganisms via the experimental adaptive evolution method; (**B**) Analysis of QAC-associated antibiotic resistance via the disk diffusion method. Created in BioRender. Seferyan, M. (2025) https://BioRender.com/tfcgi1l.

The general concern about the widespread use of QAC-based compositions is the possibility of the spreading of associated antibiotic resistance due to uncontrolled QAC exposure to bacterial colonies in the environment [63,64]. Co-resistance occurs when QAC resistance genes (such as qac genes) are genetically linked on the same mobile genetic elements—plasmids, integrons, or transposons—that also carry antibiotic resistance genes. This means that when bacteria develop resistance to QACs, they may simultaneously acquire antibiotic resistance genes due to this genetic linkage [65,66]. A second, similar type of bacterial survival strategy is the cross-resistance phenomenon, where mechanisms such as efflux pumps, changes in membrane structure, or genetic mutations that confer resistance to QACs simultaneously affect antibiotics with similar targets or modes of action [67,68]. Herein, we conducted a two-step study, including long-term resistance selection to QACs, followed by an antibiotic activity test on acquired strains to evaluate the possible co- and/or cross-resistance (Figure 6).

The assay was conducted on four reference strains including *S. aureus* ATCC 43300, *E. coli* ATCC 25922, *K. pneumoniae* ATCC 70060, and *A. baumannii* ATCC 15308. The main objects of the study were the two lead QACs obtained earlier **1,6-NQ-9** and its structural isomer **2,7-NQ-9**, for comparison with the commercial bis-QAC **OCT**. For the analysis of antibiotic resistance, four antibiotics were used: Vancomycin (**Va30**), Colistin (**Cl10**), Bacitracin (**B10**), and Polymyxin B (**PB300**). Antibiotics were selected based on their similar molecular targets and mechanisms of action [69,70,71,72]. The bacterial resistance evaluation for **2,7-NQ-9** and **OCT** was conducted earlier and is represented for comparison.

**Figure 7:**
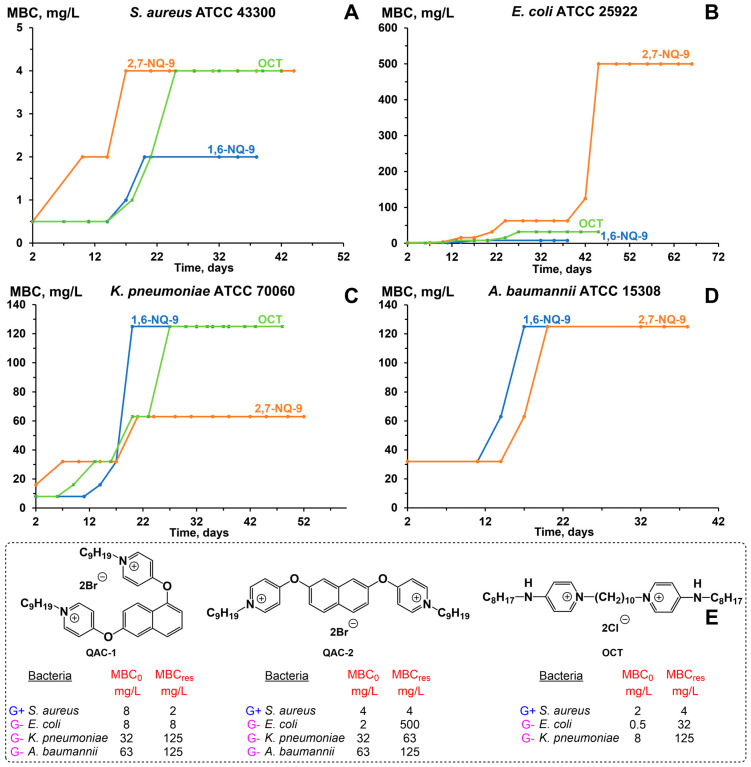
Bacterial resistance study of hit bis-QACs (**E**) in strains of *S. aureus* ATCC 43300 (**A**), *E. coli* ATCC 25922 (**B**), *K. pneumoniae* ATCC 700603 (**C**), and *A. baumannii* ATCC 15308 (**D**).

Firstly, the selection of the QAC-resistant microorganisms was carried out via the experimental adaptive evolution method, conducted at stepwise increasing concentrations of QACs over a prolonged period of time (Figure 7). The results were recorded based on the change in the MBC_res_ values compared to the initial MBC_0_ (Figure 6). Overall, **1,6-NQ- 9** and **2,7-NQ-9** showed slightly different resistance patterns. MBC values for *S. aureus* and *A. baumannii* did not show a significant increase, retaining optimal activity within one dilution during 42 days of continuous biocide exposure (Figure 7A,D). Noticeably, the effect on *A. baumannii* was expressed almost identically for both cationic biocides. A slight decrease in efficiency against *K. pneumoniae* was evident for **1,6-NQ-9** after 20 days of the experiment, whereas **2,7-NQ-9** showed similar, but not as extensive MBC growth (Figure 7C). Modest dissimilarities in pathogen evolution were observed in the *E. coli* strain (Figure 7B). While the activity values for **QAC-2** decreased significantly after 40 days with a 250-fold difference compared to MBC_0_, the MBC level of **QAC-1** remained at a relatively similar level for almost 50 days, demonstrating the initial preservation of efficiency with a consequential four-fold MBC growth towards the end of the experiment. The outlined differences suggest that even minor conformational changes in the structures of the cationic biocides can affect the level and rate of the development of bacterial resistance. It is also worth noting that for most of the described cases, the lead compound **1,6-NQ-9** was not inferior to the commercial antiseptic **OCT**. Additionally, we conducted a fungal resistance study on the *C. auris* DSM 21092 strain for **1,6-NQ-9** and **2,7-NQ-9** (Figure S2). The obtained patterns were similar to that of Gram-positive *S. aureus,* without resistance development within 47 days.

**Figure 8:**
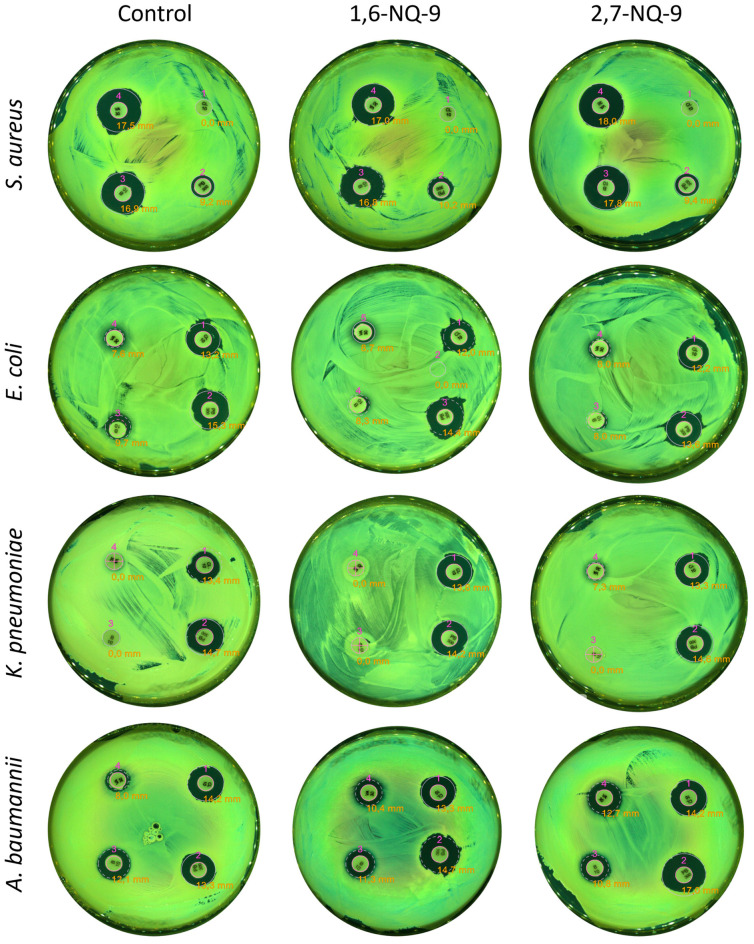
Analysis of QAC-associated antibiotic resistance via the disk diffusion method. Plates were imaged using the Interscience Scan 500 instrument (Saint-Nom-la-Bretèche, France) under optimized illumination, and inhibition zones were automatically measured with the integrated Scan software (version 3.4).

Then, eight bacterial forms obtained after selection with **1,6-NQ-9** and **2,7-NQ-9** were analyzed for antibiotic resistance using the disk diffusion method (Figure 6B). The activity values (inhibition zones) of antibiotics against the original pathogens before mutation were used as a control (Table S5, Figure 8). To our surprise, the antibacterial efficacy of the designated antibiotics remained unchanged. On the contrary, a modest growth in the inhibition zones was observed in some cases. Thus, **Va30** showed greater activity against *A. baumannii* ATCC 15308, where the inhibition zone increased by 4.1 mm. A similar picture was observed for **PB300**, where an increase of 3 mm occurred compared to the control on the *A. baumannii* ATCC 15308. In other cases, the changes were not significant enough and remained within the standard error of the control evaluations. Thus, co- or cross-resistance to antibiotics was not observed when using the developed QACs. While these results are encouraging, we would like to emphasize that this topic requires further attention from the scientific community.

## 3. Materials and Methods

### 3.1. Chemistry

#### 3.1.1. General Information

All starting reagents were obtained from commercial suppliers and were used without further purification. All solvents used were reagent-grade and distilled prior to application. Air-sensitive reactions were performed under high purity argon atmosphere. Thin-layer chromatography was performed using silica gel 60 F254 aluminum sheets under UV light. 1H NMR spectra were recorded with a Bruker AM300 instrument (Billerica, MA, USA) at ambient temperature in dimethyl sulfoxide-d_6_ (DMSO-d_6_).

#### 3.1.2. Tested Compounds

Compounds **CPC**, **OCT**, **1,6-NQ-9,** and **2,7-NQ-9** were reproduced for the microbiological activity assay of biocide compositions and resistance study. Full experimental and characterization data for **1,6-NQ-9** and **2,7-NQ-9** can be found elsewhere [40,73]. **CPC** was synthesized from pyridine using the widely known method of N-alkylation by cetylchloride in dry acetonitrile. **OCT** was synthesized using a previously described method [39].

#### 3.1.3. Synthesis Procedure and Characterization of 1,6-NQ-9 Precursor-4,4′-(naphthalene-1,6-diylbis(oxy))dipyridine

1,6-Dihydroxynaphtalene (208 mg, 1.3 mmol, 1 eq.), cesium carbonate (2.54 g, 7.8 mmol, 6 eq.), and copper (I) iodide (59 mg, 0.312 mmol, 0.24 eq.) were placed in an oven-dried 10 mL flask equipped with a magnetic stirrer. The flask was then evacuated and back-filled with argon (the procedure was repeated three times). Then 4-chloropyridine hydrochloride (468 mg, 3.12 mmol, 2.4 eq.) and picolinic acid (123 mg, 0.624 mmol, 0.48 eq.) were added to the mixture, followed by 2 mL DMSO_dry_. The flask was re-evacuated and back-filled with argon again (the procedure was repeated three times), then placed in the oil bath and heated to 120 °C. The reaction mixture was stirred vigorously for 48 h and monitored by thin-layer chromatography (hexane: ethylacetate = 1:1, R_f_ = 0.7). After cooling to room temperature, the reaction mixture was diluted with 10 mL of EtOAc, filtered, and the organic phase was concentrated in vacuo. The obtained crude product was extracted in refluxing hexane, then filtered, evaporated, and dried under high-vacuum conditions, giving the desired product.

White solid (0.327 g, 1.04 mmol, 80% yield). **C_20_H_14_N_2_O_2_, M =** 314.34; **^1^H NMR** (300 MHz, DMSO-*d*6): δ 8.52–8.41 (m, 4H, 4CHPyr), 7.97 (d, J = 9.1 Hz, 1H, 1CHAr), 7.90 (d, J = 8.0 Hz, 1H, 1CHAr), 7.83 (d, J = 2.2 Hz, 1H, 1CHAr), 7.62 (t, J = 8.0 Hz, 1H, 1CHAr), 7.40 (dd, J = 9.1 Hz, 2.2 Hz, 1H, 1CHAr), 7.33 (d, J = 8.0 Hz, 1H, 1CHAr), 7.02 (d, J = 4.9 Hz, 2H, 2CHPyr), 6.97 (d, J = 4.9 Hz, 2H, 2CHPyr).

### 3.2. Microbiological Assay

#### 3.2.1. Bacterial and Fungi Strains

Laboratory strains of the microorganisms *E. coli* ATCC 25922, *K. pneumoniae* ATCC 70060, *S. aureus* ATCC 43300, *P. aeruginosa* ATCC 27853, *A. baumannii* ATCC 15308, and *C. auris* DSM 21092, were obtained from the State Collection of Pathogenic Microorganisms (Obolensk, Russia). Clinical strains of *E. coli* B-3421/19 (urine), *E. coli* C226691/21 (urine), *K. pneumoniae* B-2523/18 (trachea), *K. pneumoniae* C24540/21 (trachea), *S. aureus* B-8648 (wound), *S. aureus* MRSA 0576 (wound), *P. aeruginosa* B-2099/18 (catheter), *P. aeruginosa* C23520/21 (catheter), *A. baumannii* B-2926/18 (catheter), *A. baumannii* C23382/21 (catheter), and *C. auris* KA9 (urethra) were isolated in the Molecular Microbiology Department of the Federal Budget Institution of the Science State Research Center for Applied Biotechnology and Microbiology (FBSI SRC PMB, Obolensk, Russia) from clinical samples in the investigation of infection cases in 2018–2021.

#### 3.2.2. Identification of Microorganisms

Species identification of the microorganisms was carried out on a MALDI-TOF Biotyper mass spectrometer (Bruker, Billerica, MA, USA) according to the standard microflex user manual. One part of the daily bacterial culture was introduced into a 2 mL tube (Eppendorf, Hamburg, Germany) containing 300 µL of deionized water and triturated until a homogeneous suspension was obtained. Then, 900 µL of 96% ethanol was added and shaken. The tube was then centrifuged at 12,000× *g* for 2 min. The supernatant was discarded, and the precipitate was dried in air. After drying, the precipitate was thoroughly mixed with 30 µL of 70% formic acid, incubated for 10 min at room temperature, and then mixed with 30 µL of acetonitrile (Sigma-Aldrich, St. Louis, MO, USA) and incubated for another 10 min. This mixture was centrifuged at 12,000× *g* for 2 min; then, 1 µL of the supernatant was transferred to an MSP 96 Polished Steel MALDI Target Plate instrument (Bruker Daltonik GmbH, Bremen, Germany), air-dried, and covered with 1 µL of a saturated solution of α-cyano-4-hydroxycinnamic acid (Bruker Daltonik, Bremen, Germany). The MALDI target plate was placed in a MALDI-TOF microflex LRF instrument (Bruker Daltonik, Bremen, Germany). The data acquisition settings were as follows: ion source 1 at 19.50 kV, ion source 2 at 18.22 kV, lens at 7.01 kV, and a mass range of 2000 to 20,000 Da. Spectrum registration was performed automatically using the MALDI Biotyper RTC 3.1 software (Bruker Daltonik, Bremen, Germany). The obtained spectra were compared with the reference spectra of the FFL v1.0 database for experimental control. Identification scores ≥2.0 and 1.7–1.99 indicated recognition of the species and genus levels, respectively, while scores <1.7 indicated a lack of reliable data.

#### 3.2.3. Cultivation of Microorganisms

Microorganisms were cultivated using agar and Mueller–Hinton broth. The cultivation of microorganisms was carried out for 20–24 h at a temperature of 37 °C.

#### 3.2.4. Determination of the Biofilms

The efficiency of bacterial biofilm formation was determined using a method based on the ability of the crystal violet dye to bind with cells and the biofilm matrix. The growth of biofilms of the test strains of microorganisms was carried out in 96-well plates, according to the standard method [74]. To obtain biofilms, 96-well flat-bottomed culture plates were used. In each well, 200 µL of a daily bacterial culture was inoculated at a concentration of 10^6^ CFU/mL and cultivated for 24 h at 37 °C. Then, the medium with planktonic cells was carefully taken from the plate. Wells with biofilms were washed for 2–3 min using sterile PBS buffer (NaCl—8 g, KCl—0.2 g, Na_2_HPO_4_—1.44 g, KH_2_PO_4_—0.24 g per 1 L, pH = 7.4) in the same volume to remove the residual planktonic cells. After washing, PBS was completely removed by pipetting. Then, 200 µL of a filtered 0.1% solution of gentian violet was added to each well, and the biofilms were incubated with the dye for 10–15 min at room temperature. The dyes were completely removed from the well by pipetting. The unbound dye was thoroughly washed off with PBS. The plates were inverted onto filter paper and dried. After complete drying of the surface, a 200 µL mixture of ethanol–isopropanol (1:1) was added to the wells. The dye was washed off from the surface of the wells, taken, and placed in clean flat-bottomed plates. The optical density of the resulting solution was measured at a wavelength of 590 nm using an xMark™ Microplate Absorbance Spectrophotometer (Bio-Rad Laboratories Inc., Hercules, CA, USA). The measurement results were interpreted by comparing the OD_590_ values of the samples with control values (pure solvent without dye added).

The absence of biofilm was recorded at OD_590_ sample ≤ OD_590_ control; a weak degree of biofilm production at OD_590_ (control) < OD_590_ (sample) ≤ 2*OD_590_ (control); an average degree of biofilm production at 2*OD_590_ (control) < OD_590_ (sample) ≤ 4*OD_590_ (control); and a high degree of biofilm production at OD_590_ control < 4*OD_590_ sample [75]. All experiments were carried out in triplicate.

#### 3.2.5. Antimicrobial and Antibiofilm Activity

For the alcohol-based composition activity test, QACs were dissolved in the designated alcohol mixtures (2-phenoxyethan-1-ol/water (solution 2%, *v*/*v*), isopropyl alcohol/water (solution 63.5%, *v*/*v*), isopropyl alcohol/propyl alcohol/water (1/2/2, *v*/*v*/*v*)) with a final combined concentration of 1 mg/mL. The samples were treated by sonication for 15 min at 25 °C to improve dissolution. For the QAC + QAC synergy test, QAC mixtures of a 1/1 ratio (**CPC + OCT**, **CPC + 1,6-NQ-9**, **OCT + 1,6-NQ-9**) were dissolved in the DMSO/water system at a ratio of 1/9, with a final combined concentration of 1000 mg/L. For better dissolution, the samples were sonicated for 15–60 min at 40 °C. The MIC and MBC of the antimicrobials was determined by the microdilution method in culture broth, as indicated by the Clinical and Laboratory Standards Institute of the United States of America [76]. For this, two-fold dilutions of the tested solutions (500–0.25 mg/L) were prepared in nutrient broth.

Preparation of the initial bacterial suspension. From single colonies grown in Mueller–Hinton broth at 37 °C for 18 h, a suspension with an optical density of 0.5 according to the McFarland standard in sterile saline was prepared, which corresponds to approximately 1–2 × 10^8^ CFU/mL. The suspension was then diluted 100-fold by adding 0.2 mL of the suspension to a flask containing 19.8 mL of nutrient Mueller–Hinton broth. The concentration of microorganisms in this case was 10^6^ CFU/mL.

Study of planktonic cells of microorganisms. Nutrient broth with the tested biocide compositions (0.1 mL) was added to 12 wells in horizontal rows of the culture plate. Nutrient broth without biocide compositions was added to separate rows for the control. Next, 0.1 mL of the original suspension was added to the wells with the test preparation and the control wells with broth. The final concentration of microorganisms in each well was 5 × 10^5^ CFU/mL. The plates were covered with lids and placed in a thermostat (37 °C) for 20 h. MIC was determined visually, by the presence of bacterial growth/turbidity in the well. The MBC was determined based on the results of seeding on solid nutrient media. For this purpose, 10 μL of each well in which there was no visible growth (by the presence of turbidity) were seeded on Mueller–Hinton agar. The results were taken into account based on the presence of culture growth at the site of application after 48 h of incubation at 37 °C. If there was no growth in a well, but growth of the studied culture was observed when seeding from this well onto a solid nutrient medium, this concentration was taken as the MIC. In this case, the lowest concentration at which cell growth was completely suppressed when seeding onto a solid nutrient medium was taken as the MBC. Since the detection limit for this method is 10 CFU/mL, the absence of any growth on a plate with Mueller–Hinton agar indicates that the concentration is below this value. Thus, the initial concentration of 10^5^ CFU/mL was reduced to a level below 10 CFU/mL. Therefore, the MBC was considered the minimum concentration of the antimicrobial agent capable of inactivating more than 99.99% of the bacteria present. Three experiments were conducted for each strain and antimicrobial compound.

Study of microbial biofilms. Next, 0.2 mL of the initial bacterial suspension was added to the wells of the culture plate, as described above, at a concentration of 5 × 10^6^ CFU/mL. The plates were covered with lids and placed in a thermostat (37 °C) for 20 h. After some time, the medium with planktonic cells was carefully taken from the wells of the plate, and the wells with biofilms were washed for 2–3 min with sterile PBS buffer (NaCl—8 g, KCl—0.2 g, Na_2_HPO_4_—1.44 g, KH_2_PO_4_—0.24 g per 1 L, pH = 7.4) in the same volume, after which, the buffer was completely removed by pipetting. Next, two-fold dilutions of the nutrient broth with the tested QACs (0.2 mL) were added to 12 wells in horizontal rows of the culture plate with washed biofilms. Nutrient broth without biocide compositions was added to separate rows for the control. The plates were covered with lids and placed in a thermostat (37 °C) for 20 h. MBIC was determined visually, by the presence of bacterial growth/turbidity in the well. MBEC was determined based on the results of sowing on solid nutrient media. For this purpose, 10 μL of each well in which there was no visible growth (based on turbidity) were plated on Mueller–Hinton agar. The results were taken into account based on the presence of culture growth at the site of application after 48 h of incubation at 37 °C. If there was no growth in a well, but growth of the studied culture was observed when plated from this well onto a solid nutrient medium, this concentration was taken as the MBIC. In this case, the lowest concentration at which cell growth was completely suppressed when plated onto a solid nutrient medium was taken as the MBEC. Since the detection limit for this method is 10 CFU/mL, the absence of any growth on a plate with Mueller–Hinton agar indicates that the concentration is below this value. Thus, the initial concentration of 10^5^ CFU/mL was reduced to a level below 10 CFU/mL. Therefore, the MBEC was considered the minimum concentration of an antimicrobial agent capable of inactivating more than 99.99% of the bacteria present. Three experiments were performed for each strain and antimicrobial composition.

#### 3.2.6. Checkerboard Assay

An investigation into the synergistic effect of QACs was conducted on planktonic and biofilm cultures of the studied bacterial strains. Preparation of the initial bacterial suspension was conducted in the same way as in Section 3.2.5.

Study of planktonic bacterial cells. Working solutions were prepared using nutrient broth as a solvent. For the study, 3–5 mL of working solutions with concentrations of 8 × MBC of substances X and Y were prepared. To dilute substance X, a working plate in which the study was conducted was used. A total of 100 μL of working solution X was added to each well of row A of the working plate. Next, 50 μL of nutrient broth was added to the wells of rows B-H. Using an eight-channel dispenser, 50 μL of the resulting solutions were successively mixed and transferred from well row A to well row G. As a result, two-fold serially decreasing dilutions of substance X were obtained. Next, 50 μL of liquid was removed from well row G. The wells of row H did not contain substance X for the control of the activity of substance Y. An additional plate was used to dilute substance Y. Subsequently, 120 μL of the working solution of the substance was added to the wells of column 12 of the additional plate, and 60 μL of nutrient broth was added to the wells of columns 1–11. Next, 60 μL of the obtained solutions were successively mixed and transferred from column 11 to column 2. Thus, two-fold serially decreasing dilutions of substance Y were obtained. The wells of column 12 did not contain substance Y for the control of the activity of substance X.

Next, 50 μL was transferred from all wells of the additional plate to the same wells of the working plate. Then, 100 μL of the pre-prepared initial bacterial suspension of the studied strain at a concentration of 10^6^ CFU/mL was added to all wells of the working plate. Upon the completion of all actions, the volume of liquid in each of the 96 wells in the working plate was 200 μL (50 μL of X + 50 μL of Y + 100 μL of nutrient medium containing 10^6^ CFU/mL of the culture of the studied strain). Nutrient broth in well H12 without biocide compositions was used to control microbial growth. The plates were covered with lids and placed in a thermostat (37 °C) for 20 h.

MIC was determined visually by the presence of bacterial growth/turbidity in the well. MBC was determined based on the results of seeding on solid nutrient media. For this purpose, 10 μL of each well in which there was no visible growth (based on the presence of turbidity) were seeded on Mueller–Hinton agar. The results were taken into account based on the presence of culture growth at the site of application after 24 h of incubation at 37 °C. If there was no growth in the well, but growth of the studied culture was observed when seeding from this well on a solid nutrient medium, this concentration was taken as MIC. In this case, the lowest concentration at which cell growth was completely suppressed when seeding on a solid nutrient medium was taken as MBC.

Cultivation of bacterial biofilms. A volume of 0.2 mL of the pre-prepared initial bacterial suspension, as described above, at a concentration of 10^6^ CFU/mL was added to the wells of the culture plate. The plates were covered with lids and placed in a thermostat (37 °C) for 20 h. After some time, the medium with planktonic cells was carefully taken from the wells of the plate, and the wells with biofilms were washed for 2–3 min with sterile PBS buffer (NaCl—8 g, KCl—0.2 g, Na2HPO4—1.44 g, KH_2_PO_4_—0.24 g per 1 L, pH = 7.4) in the same volume, after which, the buffer was completely removed by pipetting.

Study of biofilms. Working solutions were prepared using nutrient broth as a solvent. For the study, 3–5 mL of working solutions with concentrations of 4 × MBC of substances X and Y were prepared. Separate plates 1 and 2 were used for dilution and 220 μL of working solution X was added to each well of row A of plate 1. Next, 100 μL of nutrient broth was added to the wells of rows B-H. Using an eight-channel dispenser, 110 μL of the obtained solutions were successively mixed and transferred from well row A to well row G. As a result, two-fold serially decreasing dilutions of substance X were obtained. Next, 110 μL of liquid was removed from well row G. The wells of row H did not contain substance X for the control of the activity of substance Y.

A volume of 220 μL of the working solution of the substance was added to the wells of column 11 of plate 2, 110 μL of nutrient broth was added to the wells of columns 1–11. Next, 110 µL of the resulting solutions were successively mixed and transferred from column 11 to column 2. In this way, two-fold serially decreasing dilutions of substance Y were obtained. The wells of column 12 did not contain substance Y for the control of the activity of substance X.

Next, 100 μL were transferred from all the wells of plates 1 and 2 to the same wells of the plate with washed biofilm. Upon the completion of all actions, the volume of liquid in each of the 96 wells of the plate with biofilm was 200 μL (100 μL X + 100 μL Y). Nutrient broth in well H12 without biocide compositions was used to control microbial growth. The plates were covered with lids and placed in a thermostat (37 °C) for 20 h.

MBIC was determined visually, by the presence of bacterial growth/turbidity in the well. MBEC was determined based on the results of seeding on solid nutrient media. For this, 10 μL was seeded on Mueller–Hinton agar from all wells in which there was no visible growth (by the presence of turbidity). The results were taken into account by the presence of culture growth at the site of application after 48 h of incubation at 37 °C. If there was no growth in the well, but growth of the studied culture was observed when seeding from this well onto a solid nutrient medium, then this concentration was taken as the MBIC. In this case, the lowest concentration at which cell growth was completely suppressed when seeding onto a solid nutrient medium was taken as the MBEC.

Calculation of indices. Indices calculation points were chosen based on the use of the minimum active concentrations of individual components in the mixture The FICI, FBCI, FBICI, and FBECI were calculated for QAC combinations by the following formulas:

FICI (X + Y) = (MIC_X_ (comb.)/MIC_X_ (alone)) + (MIC_Y_ (comb.)/MIC_Y_ (alone))

where

MIC (comb.)—MIC value of components in combination
MIC (alone)—MIC value of individual components

FBICI (X + Y) = (MBIC_X_ (comb.)/MBIC_X_ (alone)) + (MBIC_Y_ (comb.)/MBIC_Y_ (alone))

where

MBIC (comb.)—MBIC value of components in combination
MBIC (alone)—MBIC value of individual components

FBCI (X + Y) = (MBC_X_ (comb.)/MBC_X_ (alone)) + (MBC_Y_ (comb.)/MBC_Y_ (alone))

where

MBC (comb.)—MBC value of components in combination
MBC (alone)—MBC value of individual components

FBECI (X + Y) = (MBEC_X_ (comb.)/MBEC_X_ (alone)) + (MBEC_Y_ (comb.)/MBEC_Y_ (alone))

where

MBEC (comb.)—MBEC value of components in combination
MBEC (alone)—MBEC value of individual components

The FICI, FBCI, FBICI, and FBECI were interpreted as follows: index ≤ 0.5—the combination is more effective than either drug alone, i.e., synergistic; 0.5 < index ≤ 2.0—the combination’s effect is additive; 2.0 < index ≤ 4.0—the combination’s effect is indifferent, and index > 4—the combination’s effect is antagonistic.

#### 3.2.7. Time–Kill Kinetics

Determination of the time–kill kinetics was performed on *S. aureus* ATCC 43300. The antibacterial activity of the compounds and compositions was assessed in an experiment to study the survival of bacterial cells in MHB containing the test compounds at a concentration equal to the minimum bactericidal concentration (MBC) at different exposure times. MHB was prepared with appropriate additions of hit-compounds. During the study, different time intervals for exposure to compounds and compositions were selected, corresponding to 1 min, 3 min, 5 min, 10 min, 15 min, 30 min, 45 min, 60 min, 75 min, 90 min, 105 min, and 120 min for each strain under study. The test microorganism in logarithmic growth phase was added to each tube containing 4 mL of MHB and the test compound at the required concentration. The final concentration of microbial cells in the vial was 5 × 10^5^ CFU/mL. The tubes were incubated at 37 °C. At certain cultivation time intervals, aliquots of the suspension (100 μL) were taken, 5 successive 10-fold dilutions of the selected suspension were made, and 0.1 mL was inoculated onto a solid nutrient medium to determine the concentration of microbial cells in the test tube. All studies were carried out in triplicate, and test tubes without the addition of the compounds under study were used as a negative control. The incubation of bacteria on dishes with a dense nutrient medium was carried out at a temperature of 37 °C for 24 h. For the studied strains, a concentration of compounds equal to the MBC for each compound was chosen.

#### 3.2.8. Microbial Resistance Assay

The selection of resistant microorganisms is carried out according to the previously proposed method of experimental adaptive evolution, by regular culture of the bacterial strains in a nutrient broth containing serial dilutions of the antibacterial drug [77,78,79]. To do this, in 2 mL of nutrient Mueller–Hinton broth, a series of two-fold dilutions of the drug were prepared, starting with a concentration that was half of the MIC. During the initial inoculation, 20 µL of 10^7^ CFU/mL of daily culture was added to each tube and incubated at 37 °C. After two to three days of incubation, 50 µL from the last turbid tube with a higher concentration was divided into new tubes with increasing concentrations of the tested antibacterial drug. During subsequent transfers to increasing concentrations, 50 µL was also taken from the last tube, in ascending order, in which visual growth was observed. The selection process was stopped if the MBC had not changed within 6–8 passages. The MBC was determined according to method in Section 3.2.5.

#### 3.2.9. Disk Diffusion Method (Co-Resistance to Antibiotics)

The antibiotic sensitivity of bacterial strains obtained by selective selection in the presence of QACs was determined by the disk diffusion method. For this purpose, a lawn of 100 μL of bacterial suspension equivalent to a turbidity standard of 0.5 according to McFarland and containing approximately 1.0–2.0 × 10^8^ CFU/mL of bacterial culture was seeded onto the surface of a dense Mueller–Hinton agar. The dishes were left for 15 min until the suspension was completely absorbed. Then, sterile cellulose disks containing certain concentrations of the antibiotics under study were aseptically applied to the agar surface. The crops were incubated in a thermostat at a temperature of 37 °C for 18–24 h. After incubation, the results were recorded by measuring the diameter of the growth inhibition zone of the bacterial culture around the disk in millimeters. The results were analyzed using data from the European Committee on Antimicrobial Susceptibility Testing (EUCAST). A Petri dish containing the microbial culture was placed in the illuminated observation chamber of the Interscience Scan 500 counter to ensure optimal lighting and image visualization. The images were then processed either automatically or semi-automatically using the integrated Scan software (version 3.4), which enabled the precise measurement of microbial growth inhibition zone diameters. The Scan software is designed to quantify colonies and antibiotic inhibition zones using the automated Interscience Scan 500 counter. The antibiotic sensitivity of reference bacterial strains before selection was used as the control.

## 4. Conclusions

In summary, we have conducted a broad microbiological assay of QAC-based biocide compositions. The resulting study model allowed us to evaluate the different interactions within antimicrobial mixtures and highlight key aspects. As a result of the analysis, it was confirmed that combining QACs with alcohols increased the activity profile of QACs against bacterial pathogens. This effect was especially evident on biofilm cultures, where an up to 64-fold MBIC and 32-fold MBEC reduction was registered. Additional microbiological studies on an expanded panel of microorganisms showed the ability of designated compositions to exhibit antifungal action on yeast-like *C. auris*, including multi-resistant strains. This is further confirmation of the effectiveness of this approach, suggesting that the mechanisms of antimicrobial action of QACs and alcohols are complementary. Next, we performed an optimized checkerboard assay combining two indicators of synergistic effect—inhibitory and bactericidal—for planktonic cells as well as biofilms. The proposed approach allowed us to hypothesize the validity of partial synergy in the presence of only one of the effects. Although we were not able to confirm the initial hypothesis that combinations of mono- and bis-QACs would exhibit a synergistic effect on Gram-negative strains and most of the interactions were additive, a synergy of QAC mixtures on Gram-positive *S. aureus* was detected. The bactericidal profile of substances with FBCI 0.39 was noticeably affected. While it can be said that the basic paradigm, which assumes additivity as the main interaction between QACs, is true for most cases, the obtained results suggest that intra-class synergies also exist and require further study. Moreover, the optimized checkerboard assay for biofilm cultures revealed the synergy of QAC combinations on *A. baumannii*. Therefore, special attention should be directed toward the scarcely investigated topic of the synergistic effect of antibacterial agents on biofilms. The resistance assay of lead bis-QACs via the experimental adaptive evolution method showed promising results, where three out of five strains did not acquire high levels of resistance to the QACs over a long period of exposure. QACs demonstrated generally similar resistance patterns with slight differences in the level and rate of bacterial resistance development on *E. coli*. The obtained results are encouraging, especially considering that similar effects were observed earlier in our lab for other pyridinium-based bis-QACs. Moreover, contrary to general concerns regarding the QAC-associated spread of antibiotic resistance, we were not able to detect any cross-resistance between lead QACs and the four tested antibiotics. The presented data can be used as a framework for further microbiological and synthesis studies of bis-QACs as broad-spectrum antimicrobial agents with low resistance profiles and possible synergistic interactions.

## Figures and Tables

**Table 1:** Antibacterial activity of QACs and QAC-based compositions.

Compounds	Reference Strains					Clinical Isolates				
	*Sa*	*Ec*	*Kp*	*Ab*	*Pa*	*Sa*	*Ec*	*Kp*	*Ab*	*Pa*
Planktonic cells (MIC/MBC [mg/L])										
Control										
**CPC**	4 ± 2.3	8 ± 4.6	63 ± 17.9	32 ± 9.2	500	2 ± 0.6	8 ± 4.6	8 ± 2.3	16	32
	16	8 ± 2.3	250	125	>500	16 ± 4.6	63	16	32 ± 9.2	125 ± 35.8
**OCT**	0.5 ± 0.3	0.5	4 ± 1.2	32	8 ± 4.6	0.5	2 ± 1.2	2	32 ± 9.2	32 ± 9.2
	2 ± 1.2	0.5 ± 0.3	8 ± 4.6	125 ± 72.2	16	2 ± 0.6	4 ± 1.2	4 ± 1.2	125	63 ± 35.8
**1,6-NQ-9**	2	8	16 ± 9.2	63	32 ± 18.5	2 ± 0.6	8 ± 2.3	32 ± 9.2	125	63
	8 ± 4.6	8 ± 4.6	32	63 ± 18.5	63	4 ± 2.3	16	63	125 ± 95.3	63 ± 17.9
PhE *	125 ± 35.8	32	63 ± 18.5	16 ± 9.2	16 ± 4.6	250 ± 72.2	63 ± 17.9	125	16 ± 4.6	32
	500	32 ± 9.2	125	125	125	500	63	250	125 ± 72.2	125
IPA *	63	32 ± 9.2	32	16 ± 4.6	16 ± 4.6	63	32 ± 18.5	32 ± 9.2	16 ± 4.6	16 ± 4.6
	250	32 ± 18.5	63 ± 35.8	63	63 ± 18.5	250	32	125	32	63 ± 35.8
IPAP *	16 ± 9.2	32	63 ± 18.5	16 ± 4.6	16 ± 4.6	63 ± 17.9	63	125	16 ± 9.2	16 ± 9.2
	250	32 ± 9.2	125 ± 72.2	63 ± 17.9	63	250 ± 72.2	63 ± 35.8	125 ± 72.2	63	63
Compositions										
**PhE CPC**	0.5 ± 0.3	16	32	8	32 ± 9.2	0.5	16 ± 4.6	16	8 ± 2.3	63
	4	16 ± 9.2	32	8 ± 4.6	125	4 ± 1.2	32	32 ± 18.5	16 ± 4.6	125 ± 35.8
**PhE OCT**	1 ± 0.6	2	1	2 ± 0.6	2	1	2 ± 1.2	2 ± 0.6	4	2 ± 1.2
	8 ± 4.6	2 ± 1.2	1 ± 0.6	2	2 ± 1.2	8 ± 4.6	4	4 ± 2.3	8 ± 2.3	8
**PhE 1,6-NQ-9**	2 ± 0.6	8 ± 2.3	32	32 ± 9.2	32 ± 17.9	0.5	8 ± 4.6	32	8	32
	8 ± 4.6	8 ± 4.6	32 ± 9.2	32	63	8 ± 4.6	8	32 ± 9.2	32	125 ± 35.8
**IPA CPC**	2	8	32	4 ± 1.2	32 ± 18.5	0.5	16 ± 4.6	16	8 ± 2.3	32 ± 18.5
	8	16 ± 4.6	32	8 ± 4.6	63	2 ± 0.6	32	16 ± 9.2	8	63
**IPA OCT**	1 ± 0.3	4	1	2 ± 0.6	1	0.5	4 ± 3.2	2 ± 0.6	2	4 ± 1.2
	2	4 ± 2.3	1 ± 0.3	2 ± 0.6	1 ± 0.3	2 ± 0.6	4 ± 2.3	4	4 ± 2.3	4
**IPA 1,6-NQ-9**	1	4 ± 1.2	3 ± 18.52	16	16 ± 9.2	0.5 ± 0.3	4	32	16 ± 9.2	32 ± 18.5
	4	8 ± 4.6	32	16	63 ± 18.5	4 ± 1.2	4 ± 3.2	63 ± 18.5	32	63
**IPAP CPC**	1 ± 0.3	16	16	8 ± 2.3	16	0.5	8 ± 4.6	16	4 ± 1.2	16 ± 4.6
	4	16 ± 4.6	16 ± 9.2	8 ± 4.6	32 ± 9.2	1	16	16 ± 4.6	16	63 ± 35.8
**IPAP OCT**	1 ± 0.6	1	1 ± 0.8	2	2 ± 0.6	0.5 ± 0.3	2	2 ± 0.6	2	2
	4 ± 2.3	1 ± 0.3	1 ± 0.3	2 ± 1.2	2 ± 1.2	4	2 ± 0.6	8	4 ± 2.3	4 ± 3.2
**IPAP 1,6-NQ-9**	0.5	4 ± 1.2	16	16 ± 4.6	16	0.5 ± 0.3	4	16	4	16
	2 ± 1.2	4 ± 2.3	16 ± 4.6	16 ± 9.2	63 ± 17.9	1 ± 0.6	4 ± 2.3	16 ± 4.6	8 ± 4.6	32 ± 9.2

Note: *Sa*—*S. aureus* ATCC 43300 (laboratory strain), *S. aureus* B-8648 (clinical isolate); *Ec*—*E. coli* ATCC 25922 (laboratory strain), *E. coli* B-3421/19 (clinical isolate); *Kp*—*K. pneumoniae* ATCC 70060 (laboratory strain), *K. pneumoniae* B-2523/18 (clinical isolate); *Ab*—*A. baumannii* ATCC 15308 (laboratory strain), *A. baumannii* B-2926/ 18 (clinical isolate); *Pa*—*P. aeruginosa* ATCC 27853 (laboratory strain), *P. aeruginosa* B-2099/18 (clinical isolate); MIC—minimum inhibitory concentration; MBC—minimum bactericidal concentration; color indicates that composition exceeded activity values for at least two dilutions compared to individual components; color indicates that composition exceeded activity values for one dilution compared to individual components. * For combinations of alcohols, concentrations are indicated that correspond to the dilution threshold at which the given combination exhibits antibacterial activity.

**Table 2:** Antibiofilm activity of QACs and QAC-based compositions.

Compounds	Reference Strains					Clinical Isolates				
	*Sa*	*Ec*	*Kp*	*Ab*	*Pa*	*Sa*	*Ec*	*Kp*	*Ab*	*Pa*
Biofilms (MBIC/MBEC [mg/L])										
Control										
**CPC**	16 ± 9.2	16	63 ± 35.8	250 ± 144.3	500	8 ± 4.6	125 ± 72.2	63 ± 35.8	250 ± 144.3	500
	63	63 ± 35.8	250	500	>500	32 ± 9.2	250	500	500	500 ± 144.3
**OCT**	4	8 ± 2.3	16 ± 9.2	250	125 ± 72.2	4 ± 1.2	16 ± 9.2	16 ± 4.6	32 ± 17.9	250
	8 ± 2.3	16 ± 4.6	63	250 ± 144.3	500	8 ± 4.6	125 ± 35.8	125	250	250 ± 144.3
**1,6-NQ-9**	8	8 ± 4.6	63 ± 35.8	63 ± 17.9	500 ± 144.3	4 ± 1.2	8 ± 2.3	63	63 ± 17.9	500
	63 ± 17.9	8	500	250	500 ± 144.3	8 ± 2.3	16	125	125 ± 35.8	500
PhE *	32	16 ± 4.6	63	63 ± 17.9	125	63 ± 17.9	32 ± 9.2	63 ± 17.9	63	32
	63	32	125 ± 72.2	250	125 ± 35.8	125	63 ± 35.8	125 ± 35.8	125	125 ± 95.3
IPA *	32 ± 9.2	16	63	63 ± 17.9	125	63	32	63 ± 17.9	63 ± 17.9	32 ± 17.9
	63	32 ± 17.9	63 ± 17.9	250	125	125 ± 72.2	63 ± 17.9	125 ± 95.3	250	125
IPAP *	32	16 ± 4.6	63 ± 17.9	63	125 ± 35.8	32	32 ± 9.2	63 ± 17.9	63	32 ± 17.9
	63 ± 35.8	32 ± 9.2	63 ± 17.9	12 ± 35.85	125	125 ± 35.8	63	125	12 ± 72.25	125 ± 35.8
Compositions										
**PhE CPC**	4	8	32	16 ± 4.6	32	4 ± 2.3	16	16 ± 9.2	16	125 ± 35.8
	8 ± 4.6	32 ± 17.9	32 ± 17.9	32	63	16	32 ± 9.2	32	125 ± 35.8	125 ± 35.8
**PhE OCT**	1 ± 0.3	4 ± 2.3	8	16 ± 4.6	16 ± 9.2	2 ± 1.2	4	16 ± 4.6	16 ± 4.6	16 ± 4.6
	4	8	16 ± 4.6	32	63	4	16 ± 4.6	32	32	32
**PhE 1,6-NQ-9**	8 ± 4.6	8	32 ± 9.2	32 ± 9.2	32	4 ± 2.3	16 ± 4.6	32 ± 9.2	32	32 ± 9.2
	8	8 ± 2.3	32 ± 9.2	63 ± 17.9	63 ± 17.9	63	16 ± 4.6	32 ± 9.2	63 ± 17.9	63
**IPA CPC**	4 ± 1.2	8	16	63	63	4 ± 1.2	8 ± 2.3	16 ± 9.2	32 ± 9.2	32
	4	8 ± 2.3	32 ± 17.9	250 ± 144.3	125 ± 35.8	4 ± 1.2	125	32	63 ± 17.9	63 ± 17.9
**IPA OCT**	2 ± 0.6	4	4 ± 2.3	16 ± 4.6	8	2	4 ± 2.3	16 ± 9.2	16	16
	2 ± 0.6	8 ± 4.6	8	32 ± 9.2	32	4 ± 2.3	4	32	32	32 ± 17.9
**IPA 1,6-NQ-9**	4	8	16	32 ± 9.2	32 ± 17.9	4	8 ± 4.6	16 ± 4.6	32 ± 9.2	16
	4	8 ± 4.6	32 ± 9.2	32 ± 17.9	63	8 ± 4.6	63 ± 17.9	63	63	32
**IPAP CPC**	8	8	8	32 ± 17.9	32	8 ± 6.1	8	16	32	8 ± 2.3
	8 ± 6.1	16 ± 9.2	32 ± 17.9	32	63 ± 17.9	8	16 ± 4.6	32 ± 17.9	63 ± 35.8	32
**IPAP OCT**	1	2 ± 0.6	8	8 ± 2.3	16	2 ± 1.2	2 ± 0.6	16	16	16
	4 ± 2.3	4	8 ± 2.3	16	63 ± 17.9	4	8 ± 2.3	16 ± 4.6	32 ± 17.9	125 ± 35.8
**IPAP 1,6-NQ-9**	4	4 ± 1.2	8 ± 4.6	32 ± 9.2	32	4	4	16	32 ± 9.2	8
	8 ± 2.3	16 ± 4.6	16	32 ± 17.9	63 ± 35.8	8 ± 4.6	8 ± 4.6	32 ± 9.2	32	16 ± 4.6

Note: *Sa*—*S. aureus* ATCC 43300 (laboratory strain), *S. aureus* B-8648 (clinical isolate); *Ec*—*E. coli* ATCC 25922 (laboratory strain), *E. coli* B-3421/19 (clinical isolate); *Kp*—*K. pneumoniae* ATCC 70060 (laboratory strain), *K. pneumoniae* B-2523/18 (clinical isolate); *Ab*—*A. baumannii* ATCC 15308 (laboratory strain), *A. baumannii* B-2926/ 18 (clinical isolate); *Pa*—*P. aeruginosa* ATCC 27853 (laboratory strain), *P. aeruginosa* B-2099/18 (clinical isolate); MBIC—minimum biofilm inhibition concentration; MBEC—minimum biofilm eradication concentration; color indicates that composition exceeded activity values for at least two dilutions compared to individual components; color indicates that composition exceeded activity values for one dilution compared to individual components. * For combinations of alcohols, concentrations are indicated that correspond to the dilution limits at which the given combination exhibits antibiofilm activity.

**Table 3:** Fractional indices for antibacterial and antibiofilm activity of thr 1:1 ratio QAC combinations.

Combinations	Reference Strains					Clinical Isolates				
	*Sa*	*Ec*	*Kp*	*Ab*	*Pa*	*Sa*	*Ec*	*Kp*	*Ab*	*Pa*
Planktonic cells (FICI/FBCI)										
**CPC + OCT**	1.13	2.13	0.53	0.13	0.25	1.25	1.25	1.25	0.38	0.13
	0.28	2.13	8.06	0.03	1.03	0.56	0.53	19.53	0.31	0.38
**CPC + 1,6-NQ-9**	0.38	0.50	1.25	0.09	1.05	0.50	1.00	1.25	0.28	2.95
	0.19	0.50	0.56	0.10	4.47	0.31	0.63	1.25	0.31	5.97
**OCT + 1,6-NQ-9**	1.25	2.13	0.63	0.19	0.63	1.25	1.25	1.06	0.31	0.19
	0.31	34.00	0.31	0.10	1.25	1.50	1.25	0.53	0.26	0.25
Biofilms (FBICI/FBECI)										
**CPC + OCT**	0.63	0.38	0.63	0.06	0.16	0.75	0.14	0.63	0.56	0.10
	0.56	0.63	0.16	0.10	0.25	0.63	0.05	0.16	0.19	1.50
**CPC + 1,6-NQ-9**	0.38	0.75	0.51	0.16	0.25	0.75	0.53	1.00	0.32	0.13
	0.13	4.44	0.38	0.19	1.00	1.25	2.09	1.25	0.63	1.00
**OCT + 1,6-NQ-9**	0.75	0.50	0.63	0.16	0.16	1.00	0.38	0.63	0.75	0.38
	0.28	3.00	0.29	0.25	1.00	1.00	0.14	0.26	0.38	1.50

Note: *Sa*—*S. aureus* ATCC 43300 (laboratory strain), *S. aureus* B-8648 (clinical isolate); *Ec*—*E. coli* ATCC 25922 (laboratory strain), *E. coli* B-3421/19 (clinical isolate); *Kp*—*K. pneumoniae* ATCC 70060 (laboratory strain), *K. pneumoniae* B-2523/18 (clinical isolate); *Ab*—*A. baumannii* ATCC 15308 (laboratory strain), *A. baumannii* B-2926/ 18 (clinical isolate); *Pa*—*P. aeruginosa* ATCC 27853 (laboratory strain), *P. aeruginosa* B-2099/18 (clinical isolate); FICI—fractional inhibitory concentration index; FBCI—fractional bactericidal concentration index; FBICI—fractional biofilm inhibition concentration index; FBECI—fractional biofilm eradication concentration index; color indicates that the QAC combination exhibits a synergistic effect (index ≤ 0.5); color indicates that the QAC combination exhibits an additive effect (0.5 < index ≤ 2); no color indicates indifference (2 < index ≤ 4) or an antagonistic effect (index > 4).

## Data Availability

The original contributions presented in this study are included in the article/Supplementary Materials. Further inquiries can be directed to the corresponding author.

## Supplementary Materials

The following supporting information can be downloaded at https://www.mdpi.com/article/10.3390/ijms262412098/s1.

## Abbreviations

The following abbreviations are used in this manuscript:

QACs	quaternary ammonium compounds
MIC	minimum inhibitory concentration
MBC	minimum bactericidal concentration
BAC	benzalkonium chloride
CPC	cetylpyridinium chloride
OCT	octenidine dichloride
CHX	chlorohexidine
PA	propyl alcohol, propanol-1
IPA	isopropyl alcohol, propanol-2
PE	phenoxyethanol
MBIC	minimum biofilm inhibition concentration
MBEC	minimum biofilm eradication concentration
FIC	fractional inhibitory concentration
FBC	fractional bactericidal concentration
DMSO	dimethyl sulfoxide
ESKAPEE	*Enterococcus faecium*, *Staphylococcus aureus*, *Klebsiella pneumoniae*, *Acinetobacter baumannii*, *Pseudomonas aeruginosa*, *Enterobacter species,* and *Escherichia coli*
ATCC	American type culture collection
FICI	fractional inhibitory concentration index
FBCI	fractional bactericidal concentration index
FBICI	fractional biofilm inhibition concentration index
FBECI	fractional biofilm eradication concentration index
IZ	inhibition zone
Va30	Vancomycin
Cl10	Colistin
B10	Bacitracin
PB300	Polymyxin B
NMR	nuclear magnetic resonance
MRSA	methicillin-resistant *Staphylococcus aureus*
MFC	minimum fungicidal concentration
CFU	colony-forming unit
UV	ultraviolet
MALDI-TOF	matrix-assisted laser desorption ionization mass spectrometry
PBS	phosphate-buffered saline
OD	optical density
MHA	mueller–hinton nutrient agar
MHB	Mueller–hinton broth
