# Supplementary material for: In Pursuit of a Better Biocide Composition: Synergistic and Additive Effects of QAC-Based Formulations Against Planktonic and Biofilm Cultures
Source: Int J Mol Sci. 2025 Dec 16;26(24):12098. doi: 10.3390/ijms262412098 (PMC12733799; doi:10.3390/ijms262412098)
Supplement: Supplementary file 1 [file ijms-26-12098-s001.zip › ijms-3968185-supplementary.pdf]

Supporting Materials

In Pursuit of a Better Biocide Composition: Synergistic and Additive Effects of QAC-Based Formulations Against Planktonic and Biofilm Cultures.

Nikita A. Frolov[a], Mary A. Seferyan[a], Elena V. Detusheva[a,b], Elizabeth Son[b], Ilya G. Kolmakov [a,c], Anatoly N. Vereshchagin[*a]

[a] *N. D. Zelinsky Institute of Organic Chemistry, Russian Academy of Sciences, Leninsky prospect 47, Moscow 119991, Russia; vereshchagin@ioc.ac.ru (A. N. V.); nfrolov@ioc.ac.ru (N. A. F.).*

[b] *State Research Center for Applied Microbiology and Biotechnology, Obolensk, 142279 Serpukhov, Moscow Region, Russia;*

[c] *Lomonosov Moscow State University, Faculty of Chemistry, Leninskie Gory, 1-3, 119991 Moscow, Russia*

Table of contents

Figures, Schemes and Tables..2

Figures, Schemes and Tables.

Table S1. The antimicrobial and antibiofilm activity of QACs and QACs-based compositions against five additional multi-resistant Gram-positive and Gram-negative bacterial strains from clinical samples in the investigation of infection cases during COVID-19 period in 2021.

Compounds	Planktonic cells (MIC/MBC [mg/L])					Biofilms (MBIC/MBEC [mg/L])				
	MRSA	*Ec*	*Kp*	*Ab*	*Pa*	*MRSA*	*Ec*	*Kp*	*Ab*	*Pa*
Control										
**CPC**	1±0.3	32±17.9	32	16±4.6	250	4±1.2	125±35.8	250	32±17.9	250±72.2
	1	500	500	250±72.2	500	32	250	250	32±17.9	500
**1,6-NQ-9**	1±0.3	4±1.2	32±9.2	125±35.8	32±9.2	8±2.3	8± 6.1	32±9.2	250±72.2	63
	2± 0.6	4±2.3	63±17.9	250	63±35.8	16±9.2	32±17.9	250±72.2	500	63±35.8
Compositions										
**PhE CPC**	4±1.2	32±9.2	32±9.2	8	32	4±1.2	32±17.9	32	16	32±9.2
	4	32	63	16±4.6	125	8	32	63	32±9.2	63±17.9
**PhE 1,6-NQ-9**	2	16±4.6	32	16±4.6	32	8±4.6	1±4.66	32	32	32±9.2
	8±2.3	16±4.6	63±17.9	32±9.2	63±17.9	8±2.3	63	63±35.8	63	63
**IPA CPC**	1± 0.6	16	32	8±2.3	32	4±2.3	16±9.2	32	32±9.2	32±17.9
	4	32	32±9.2	8±2.3	63	4±1.2	16	32±9.2	32	250
**IPA 1,6-NQ-9**	1	8±6.1	32	16	16	4	8±6.1	32	32±9.2	16±4.6
	4	8±4.6	32±17.9	16±4.6	63	4±1.2	8	32±9.2	63	63
**IPAP CPC**	0,5±0.3	32±17.9	32	4±1.2	16	8	8± 6.1	16±9.2	32±9.2	16
	1	32	32±9.2	8	32±17.9	8	32	32	32	63±17.9
**IPAP 1,6-NQ-9**	0,5±0.3	4±2.3	32	8±6.1	16±9.2	4±1.2	4±2.3	16	32	16±4.6
	1± 0.6	4±1.2	32±9.2	8	32±17.9	4	8±2.3	16±4.6	32±9.2	32

Note: *MRSA – MRSA 0576*; *Ec – E. coli* C226691/21, *Kp – K. pneumoniae* C24540/21, *Ab – A. baumannii* C23382/21, *Pa – P. aeruginosa* C23520/21; MIC – minimum inhibitory concentration; MBC – minimum bactericidal concentration; MBIC – minimum biofilm inhibition concentration; MBEC – minimum biofilm eradication concentration; color indicates that composition exceeded activity values for at least two dilutions compare to individual components; color indicates that composition exceeded activity values for one dilutions compare to individual components.

Table S2. Antifungal activity of QACs and QACs-based compositions.

Compounds	Planktonic cells (MIC/MFC [mg/L])		Biofilms (MBIC/MBEC [mg/L])	
	C. auris F-2035	*C. auris KA9*	*C. auris F-2035*	*C. auris KA9*
Control				
**CPC**	4±1.2	4±1.2	8± 6.1	16±4.6
	8	8±2.3	32±17.9	32±9.2
**1,6-NQ-9**	4±1.2	4±2.3	4±2.3	4±1.2
	8±2.3	16±4.6	16±4.6	125±72.2
Compositions				
**PhE CPC**	2± 0.6	1	4±1.2	8±2.3
	2	2±1.2	4	16±4.6
**PhE 1,6-NQ-9**	8±6.1	8±4.6	16±9.2	16
	8±4.6	63±17.9	63	63
**IPA CPC**	1±0.3	1	2	4±1.2
	1±0.3	2±1.2	4	8
**IPA 1,6-NQ-9**	1	2	2±1.2	8± 6.1
	16	16±4.6	32±9.2	64
**IPAP CPC**	1	1±0.3	2	4±1.2
	2± 0.6	4	8±2.3	8±6.1
**IPAP 1,6-NQ-9**	0.5	1± 0.6	2±1.2	4
	2±1.2	8±2.3	16±4.6	32±9.2

Note: MIC – minimum inhibitory concentration; MFC – minimum fungicidal concentration; MBIC – minimum biofilm inhibition concentration; MBEC – minimum biofilm eradication concentration; color indicates that composition exceeded activity values for at least two dilutions compare to individual components; color indicates that composition exceeded activity values for one dilution compare to individual components;

Table S3. Fractional indices antimicrobial and antibiofilm activity of 1:1 ratio QACs combinations against five additional multi-resistant Gram-positive and Gram-negative bacterial strains from clinical samples in the investigation of infection cases during COVID-19 period in 2021.

Combinations	Planktonic cells (FICI/FBCI)					Biofilms (FBICI/FBECI)				
	MRSA	*Ec*	*Kp*	*Ab*	*Pa*	*MRSA*	*Ec*	*Kp*	*Ab*	*Pa*
**CPC+OCT**	1,00	0,56	1,13	1,25	0,51	0,50	0,27	0,16	0,75	0,19
	3,00	15,75	0,13	0,08	0,16	1,25	0,52	0,25	0,75	0,38
**CPC+1,6-NQ-9**	1,00	1,13	1,00	0,28	1,11	0,38	1,06	1,11	0,28	0,63
	1,50	1,01	0,29	0,03	0,56	0,75	8,81	0,50	0,53	4,47
**OCT+1,6-NQ-9**	1,00	1,00	1,13	2,06	2,25	0,38	1,00	0,38	0,53	0,75
	1,00	1,00	0,19	0,32	0,19	0,75	2,50	0,06	1,03	4,97

Note: *MRSA – MRSA 0576*; *Ec – E. coli* C226691/21, *Kp – K. pneumoniae* C24540/21, *Ab – A. baumannii* C23382/21, *Pa – P. aeruginosa* C23520/21; FICI – fractional inhibitory concentration index; FBCI – fractional bactericidal concentration index; FBICI – fractional biofilm inhibition concentration index; FBECI – fractional biofilm eradication concentration index; color indicates that QAC combination exhibits a synergistic effect (index≤0,5); color indicates that QAC combination exhibits an additive effect (0,5<index ≤2); color indicates that QAC combination exhibits an antagonistic effect (index>4); no color indicates indifference or no interaction (2<index ≤4).

Table S4. Fractional indices for antifungal activity of 1:1 ratio QACs combinations.

Compounds	Planktonic cells (FICI/FFCI)		Biofilms (FBICI/FBECI)	
	C. auris F-2035	*C. auris KA9*	*C. auris F-2035*	*C. auris KA9*
**CPC+OCT**	0,63	0,38	1,06	1,13
	1,13	1,50	0,25	0,19
**CPC+1,6-NQ-9**	0,50	1,00	0,75	1,25
	7,88	1,50	0,38	0,63
**OCT+1,6-NQ-9**	0,63	0,38	1,13	1,50
	1,13	2,50	0,19	0,28

Note: FICI – fractional inhibitory concentration index; FFCI – fractional fungicidal concentration index; FBICI – fractional biofilm inhibition concentration index; FBECI – fractional biofilm eradication concentration index; color indicates that QAC combination exhibits a synergistic effect (index≤0,5); color indicates that QAC combination exhibits an additive effect (0,5<index ≤2); color indicates that QAC combination exhibits an antagonistic effect (index >4); no color indicates indifference or no interaction (2<index ≤4).

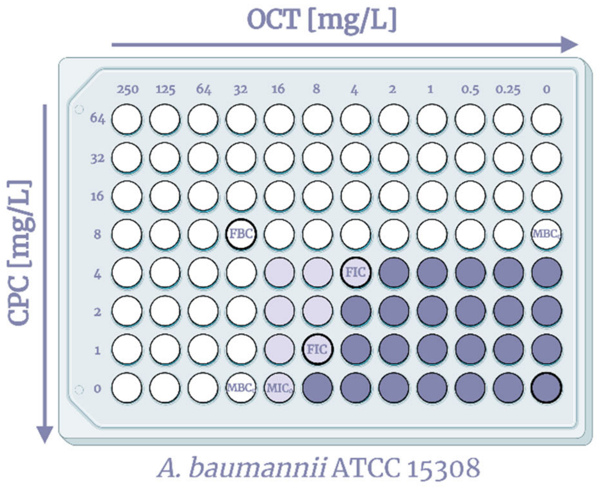

FIC$_{OCT}$	FIC$_{CPC}$	FICI	Effect
0.38±0.18	0.31±0.27	0.69±0.09	Additive
**FBC$_{OCT}$**	**FBC$_{CPC}$**	**FBCI**	**Effect**
1	1	2	Indifferent

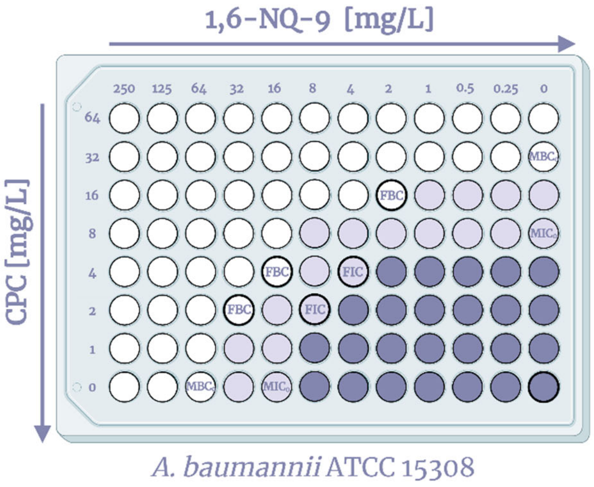

FIC$_{CPC}$	FIC$_{1,6\text{-}NQ\text{-}9}$	FICI	Effect
0.38±0.18	0.38±0.18	0.75	Additive
**FBC$_{CPC}$**	**FBC$_{1,6\text{-}NQ\text{-}9}$**	**FBCI**	**Effect**
0.23±0.2	0.26±0.23	0.49±0.1	Synergy

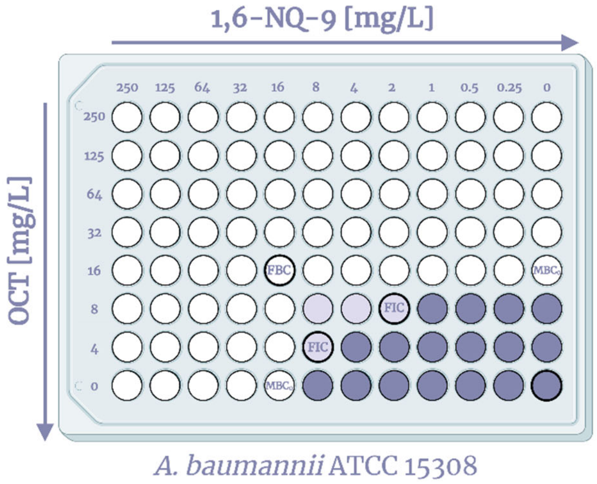

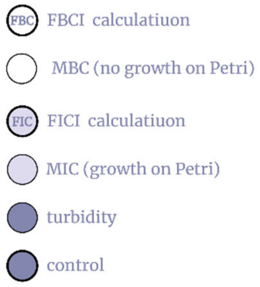

FIC$_{OCT}$	FIC$_{1,6\text{-}NQ\text{-}9}$	FICI	Effect
0.38±0.18	0.31±0.27	0.69±0.09	Additive
**FBC$_{OCT}$**	**FBC$_{1,6\text{-}NQ\text{-}9}$**	**FBCI**	**Effect**
1	1	2	Indifferent

Figure S1. Visualization of Checkerboard assay on *A. baumannii* ATCC 15308. Created in BioRender. Seferyan, M. (2025) https://BioRender.com/b2aj39l

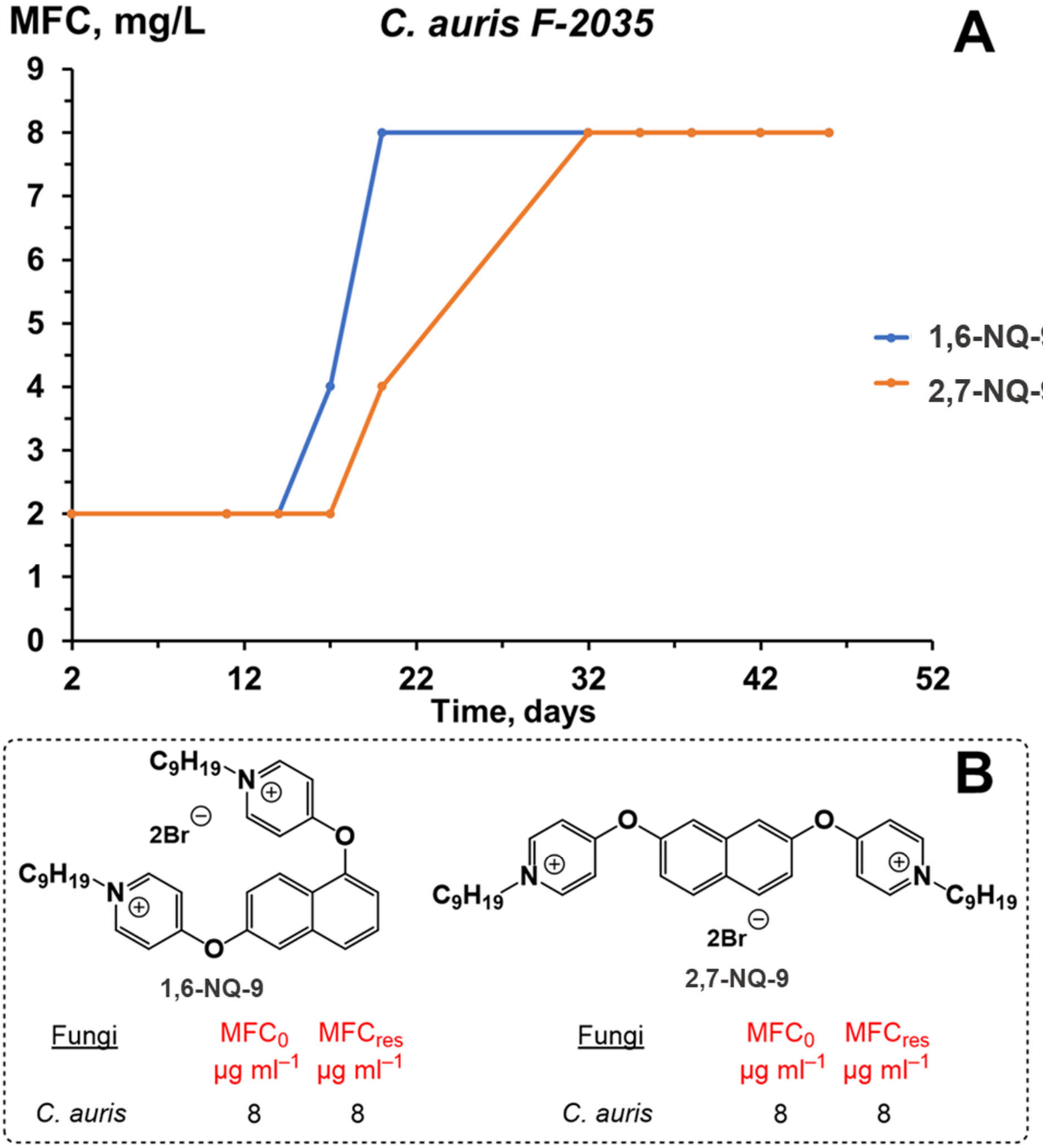

Figure S2. Fungal resistance study to hit bis-QACs (B) in strain of C. auris F-2035 (A).

Table S5. Comparison of antibiotic's inhibition zones on control and resistant bacterial strains.

Strains	Type	Antibiotics inhibition zones, mm			
		Va30 (30 µg)	*Cl10 (10 µg)*	*B10 (10U)*	*PB300 (300U)*
**S. aureus ATCC 43300**	Control	17,5	0	16,9	9,2
	1,6-NQ-9	16,9	0	16,8	10,2
	2,7-NQ-9	17,8	0	17,8	9,4
**E. coli ATCC 25922**	Control	7,6	13,5	9,5	14,8
	1,6-NQ-9	8,7	12,0	8,3	14,4
	2,7-NQ-9	8,0	12,2	8,0	13,6
**K. pneumoniae ATCC 70060**	Control	0	13,4	0	14,7
	1,6-NQ-9	0	13,8	0	14,2
	2,7-NQ-9	7,3	13,3	0	14,6
**A. baumannii ATCC 15308**	Control	8,0	14,2	12,1	13,3
	1,6-NQ-9	10,4	13,3	11,3	14,7
	2,7-NQ-9	12,1	13,5	10,8	16,3

Note: ***Va30*** – **Vancomycin**, ***Cl10*** – Colistin, ***B10*** – Bacitracin, ***PB300*** – Polymyxin B; color highlights more than 25% improvement in antibiotic activity.
